# *Rhizobiumacaciae* and *R. anhuiense* are the dominant rhizobial symbionts of *Pisum sativum* L. from Yunnan-Guizhou Plateau

**DOI:** 10.3389/fmicb.2024.1437586

**Published:** 2024-09-26

**Authors:** Junjie Zhang, Zeyang Zhao, Yufeng Feng, Jingqi Wang, Xuxiao Zong, Entao Wang

**Affiliations:** ^1^College of Food and Bioengineering, Zhengzhou University of Light Industry, Zhengzhou, China; ^2^Collaborative Innovation Center for Food Production and Safety of Henan Province, Zhengzhou University of Light Industry, Zhengzhou, China; ^3^Chinese Academy of Agricultural Sciences, Institute of Crop Sciences, Beijing, China; ^4^Departamento de Microbiología, Escuela Nacional de Ciencias Biológicas, Instituto Politécnico Nacional, Ciudad de México, Mexico

**Keywords:** *Pisum sativum*, *Rhizobium*, symbiosis, genetic diversity, *nodC* gene, symbiovar viciae

## Abstract

**Introduction:**

The aim of this study is to investigate the diversity and geographic distribution of pea-nodulating rhizobia in the subtropical region of Yunnan Province from Yunnan-Guizhou Plateau.

**Methods and results:**

A total of 615 rhizobial isolates were obtained from root nodules of the trapping plants and characterized genetically and symbiotically. The isolates discriminated into 43 genotypes by PCR-RFLP of IGS DNA. Multiple locus sequence analysis based on 16S rRNA, *recA*, *atpD, dnaK*, and *rpoB* genes placed them into eight clusters corresponding to species *R. acaciae*, *R. anhuiense*, *R. binae, R. bangladeshense, R. hidalgonense,* and three suspected novel populations of *Rhizobium* genosp. I–III. *R. acaciae* was the dominant group (52.5%) followed by *R. anhuiense* (30.7%). The other species were minor groups. Based on *nodC* phylogeny, all of them were the symbiovar viciae. All the tested strains showed efficient symbiotic N_2_ fixation on pea plants, in which WLB27, WCB18, and WNY29 presented the best PGP effects. Some of the tested strains had better IAA production, with WCB18 as the best producer (64.556 mg/L). Their distribution was mainly affected by soil available phosphorus, available potassium, and effective nitrogen. According to the results of symbiotic effect and resistance tests, strains of WLB27, WCB18, and WNY29 were selected as candidates for creating inoculants.

**Discussion:**

This suggests that the pea-nodulating rhizobia in Yunnan Province form a unique community. The results gave some novel information about the diversity, diversification, and biogeography of pea-nodulating rhizobia.

## Introduction

Pea (*Pisum sativum* L.) is one of the most significant pulse grains around the world, accounting for 16% of global pulse production due to its high protein content (up to approximately 30%) and carbohydrates for human consumption among fabaceous plants (Hacisalihoglu et al., 2020). This plant is believed to be native to the Middle and Near East, where its domestication began approximately 10,000–9,000 years B.C. It then spread progressively to other countries with settlements (Smýkal et al., 2012). Pea plant is grown worldwide over 6 million hectares in Europe, China, Canada, India, Australia, and the United States (Ning et al., 2019). This legume was introduced from Middle East to China via an ancient trade road through Southern Asia (Wu et al., 2017), and it has been widely cultivated in China for more than 2,000 years. Currently, China is the first producer of pea with a culture area of 2.4 million ha and a total production of 12.9 million tons of dry grains per year (Maluk et al., 2022).

This legume species has been recorded as a nodule-forming plant since the 1880s in North European soils (Editorinchief, 1984), and its microsymbiont, *Rhizobium leguminosarum*, was the earliest reported member of rhizobia since that time. As soil-dwelling bacteria, rhizobia are capable of fixing dinitrogen in symbiosis with legumes by inducing determined (spherical) or indetermined (rod-shaped) nodules on their roots (Poole et al., 2011). This symbiotic relationship is based on a balance of nutrient exchanges between the partners. Plants supply carbohydrates (primarily succinate and malate, products of photosynthesis) and microhabitats to the compatible bacteria, while bacteria differentiated into bacteroids in the nodules to supply ammonia (products of biological nitrogen fixation (BNF) to plant hosts) (Poole et al., 2011; Rita, 2013; Schulte et al., 2021; Rogel et al., 2011). In addition to *R. leguminosarum*, pea-nodulating rhizobia were also found in species *Rhizobium indicum* (Rahia et al., 2020), *Rhizobium ruizarguesonis* (Jorrin et al., 2020), *Rhizobium pisi* (Ramirez et al., 2008), *Rhizobium anhuiense* (Zhang et al., 2015), *Rhizobium laguerreae* (David et al., 2020), and *Sinorhizobium meliloti* (Ilahi et al., 2021), all belonging to the symbiovar (sv.) viciae (Peix et al., 2015). Except *S. meliloti, R. pisi*, *R. indicum,* and *R. anhuiense,* all the other species were closely related to the *R. leguminosarum* species, and they were grouped as the *R. leguminosarum* complex (Rlc) (Young et al., 2021). The species within the Rlc complex could only be differentiated by phylogeny of multilocus (or concatenated) sequence analysis (MLSA) of housekeeping genes but not 16S rRNA genes (Philippe et al., 2021). The presence or relative occurrence of these species varied according to the geographic areas often at region or country scales depending on soil and crop history. For instance, *R. laguerreae* was found dominant in both northwest Spain (100% of the isolates) and Tunisia (gsR, 56%) (David et al., 2020; Ilahi et al., 2021), while it formed a widespread minor group in Turkey (12.5%). In Turkey, the most common pea microsymbiont was the *R. leguminosarum* complex genospecies B (Rlc gsB, 47.5%) followed by *R. ruizarguesonis* (Rlc gsC, 20%) (Gurkanli, 2021). In addition, genospecies Rlc gsA and Rlc gsE were found as site-specific groups nodulating pea in Turkey (Gurkanli, 2021) and were not recovered in Tunisia (Ilahi et al., 2021). These previous studies demonstrated that diversity and uneven distribution of rhizobial communities associated with pea plants showed distinct geographic patterns, shaped by the adaptation of rhizobia to local conditions that may impact symbiosis interaction and functioning.

Application of chemical fertilizers has effectively enhanced crop yields, but their long-term excessive use has led to increased costs, reduced fertilizer efficiency in agricultural production, as well as severe environmental and biodiversity degradation (Drew et al., 2012). The integrated use of synthetic and bio-fertilizers is a sustainable eco-friendly agricultural approach for crop sustainability. Consequently, increasing the use of bio-fertilizers can help to reduce the demand for chemical fertilizers, which have a number of negative environmental effects (Gao et al., 2020; Ahmed et al., 2021). In recent years, the exploitation and improvement of BNF of field pea is a key element for eco-sustainable agriculture (Holt-Gimnez and Altieri, 2013) based upon its worldwide cultivation and high BNF efficiency. It was estimated that more than half of the biologically fixed nitrogen worldwide is yielded by rhizobium-legume symbioses (Vaclav, 2005). BNF in pea crops can reach one of the highest levels with more than 80% of the N provided to plants, while an average of only 25–35 kg/hm of N is introduced in the soil, depending on the tillage and cropping system (Xiaofang and Zhangqun, 2023). Therefore, isolation and phenotypic screening of rhizobial strains to select bio-inoculants is a crucial strategy. Such screening is also important because the efficiency of the symbiosis can be affected by many other factors, such as host specificity and the ability of the selected strain to compete with local rhizobia (Virginie et al., 2017). The plant growth benefits provided to the same legume species by different rhizobial strains at a given location can vary up to 10-fold (Ford and Toby, 2004).

Considering all the aforementioned aspects, and the fact that pea-nodulating rhizobia in China have not been systematically studied since the extensive revision of the *Rhizobium* and Rlc taxonomic system, we decided to conduct the present study. The aim of this study was to evaluate the diversity, relative abundance, and geographic distribution of native rhizobia that nodulate *P. sativum* in Yunnan Province, a tropic area. Traditionally, pea has been grown sparingly in Yunnan as an economic crop for fresh vegetables and grains, and the pea cultivation area has been rapidly expanded to meet the creased consumer demand. For this purpose, the taxonomic status of the isolated strains from pea root nodules was determined through ribosomal intergenic typing, phylogenies analyses of housekeeping genes (*recA*, *atpD, dnaK,* and *rpoB*), the 16S rRNA gene, and a symbiotic gene (*nodC*). In addition, soil physiochemical features were determined for estimating the distribution of rhizobia in relation to soil properties, and some plant growth promotion traits of the isolates were also characterized.

## Materials and methods

### Soil sampling and soil physiochemical characterization

Soils were sampled from 15 fields with cultivation of pea plants located in the Chuxiong, Dali, and Baoshan cities, Yunnan Province of China (Table 1). At each site, soils were sampled from a depth of 10–20 cm at the flowering stage of *Pisum sativum* in April and June 2022. For each site, five randomly taken soil subsamples of equal volume were thoroughly mixed to constitute a representative soil sample of the field site. Then, soil samples were crushed to a uniform state and transported to the laboratory in an ice-filled cooler (Zhang et al., 2018). Part of each representative soil sample was chemically analyzed for pH, conductivity (Ec), organic matter (OM), available phosphorus (AP), available potassium (AK), and effective nitrogen (EN) as described previously (Appunu et al., 2009; Han et al., 2009).

**Table 1:** Detection of the tolerance of the representative strains.

Sampling points	Strains	Acid and alkali resistance (pH)							NaCl %					Temperature °C					PEG %						Glyphosate %			
		pH = 5	pH = 6	pH = 7	pH = 8	pH = 9	pH = 10	pH = 11	0.01	1%	2%	3%	4%	4°	10°	28°	37°	45°	0%	3%	5%	7%	10%	15%	0%	0.6%	1.2%	1.8%
Chuxiong	WAB17	−	+	+	+	+	+	+	+	−	−	−	−	−	−	+	+	−	+	+	−	−	−	−	+	+	+	+
	WBY2	−	+	+	+	+	+	+	+	+	+	+	+	−	−	+	−	−	+	−	−	−	−	−	+	+	+	+
	WBY27	−	+	+	+	+	+	−	+	+	+	+	+	−	−	+	−	−	+	+	+	−	−	−	+	+	+	+
	WCB18	−	+	+	+	+	+	+	+	+	+	+	+	−	−	+	+	+	+	+	+	−	−	−	+	+	+	+
	WDB13	−	+	+	+	+	+	+	+	+	+	−	−	−	−	+	−	−	+	+	+	−	−	−	+	+	+	+
	WDY23	−	−	+	+	+	+	−	+	−	−	−	−	−	−	+	−	−	+	+	+	−	−	−	+	+	+	+
	WFY14	−	+	+	+	+	+	−	+	+	+	+	+	−	−	+	+		+	+	−	−	−	−	+	+	+	+
	WFY21	−	+	+	+	+	−	−	+	+	+	+	−	−	−	+	−	−	+	−	−	−	−	−	+	+	+	+
	WGY20	−	+	+	+	+	+	−	+	+	+	+	+	−	−	+	−	−	+	+	+	−	−	−	+	+	+	+
	WHY29	−	+	+	+	+	+	+	+	−	−	−	−	−	−	+	+	+	+	+	−	−	−	−	+	+	+	+
	WIB31	−	−	+	+	+	+	+	+	−	−	−	−	−	−	+	+	+	+	+	+	−	−	−	+	+	+	+
Dali	WJB4	−	−	+	+	+	+	+	+	+	−	−	−	−	−	+	−	−	+	+	−	−	−	−	+	+	+	+
	WJY2	−	+	+	+	+	+	−	+	+	−	−	−	−	−	+	+	−	+	+	−	−	−	−	+	+	+	+
	WKY15	−	+	+	+	+	+	+	+	+	−	−	−	−	−	+	+	−	+	+	+	+	−	−	+	+	+	+
	WKY7	−	+	+	+	+	+	+	+	+	−	−	−	−	−	+	+	+	+	+	+	+	−	−	+	+	+	+
	WLB27	−	−	+	+	+	+	+	+	+	−	−	−	−	−	+	+	−	+	+	+	+	−	−	+	+	+	+
Baoshan	WMB11	−	+	+	+	+	+	+	+	−	−	−	−	−	−	+	+	−	+	+	+	+	−	−	+	+	+	+
	WNY22	−	+	+	+	+	−	−	+	−	−	−	−	−	−	+	+	−	+	+	+	−	−	−	+	+	+	+
	WNY29	−	+	+	+	+	+	+	+	+	+	−	−	−	−	+	−	−	+	+	+	+	−	−	+	+	+	+
	WOY25	−	+	+	+	+	+	+	+	−	−	−	−	−	−	+	−	−	+	+	+	+	−	−	+	+	+	+

### Rhizobial isolation and conservation

Surface-sterilized seeds (2.5% w/v NaClO solution for 5 min) of pea varieties B and Y which referred to the varieties of Yunwan No. 18 and Yunwan No. 1, respectively, the two local varieties, were germinated, and the seeding was sown in surface-sterilized plastic pots (15 cm high × 10 cm diameter) filled with a soil-sterilized vermiculite mixture (1/5 vol/vol) as described previously (Zhang et al., 2019). All plants were grown under greenhouse conditions of 25/20°C (day/night) with a 16-h photoperiod. Sterilized water was added to the pots throughout the experiment as required. After 45 days, all plants in all soils were up-rooted, nodule numbers on each plants were counted, and bacterial strains were isolated from nodules according to the standard protocol (Zhang et al., 2019). Five plants from each soil treatment were selected for the collection of root nodules and isolation of rhizobia. The individual sterilized root nodule was crushed in sterile water, and the bacterial suspension was streaked onto a yeast extract-mannitol agar (YMA) plate. After incubation at 28°C for 3 to 5 days, single colonies representing the dominant bacteria in each plate were picked up and purified by cross-streaking on new YMA plates until pure cultures were obtained. All purified isolates were conserved in tryptone yeast (TY) solid medium broth (tryptone 5 g; yeast extract 3 g; CaCl_2_ 0.6 g; distilled water 1 L, pH 7.0) supplied with glycerol (20%, v/v) at −80°C for long-term storage and on YMA slants at 4°C for temporary storage.

### Genomic fingerprinting of rhizobial isolates

Genomic DNA (gDNA) of all rhizobial isolate was extracted according to Terefework et al. (2001). The gDNA was estimated qualitatively and quantitatively by using a NanoDrop (Thermo Fisher), and high-quality gDNA was used as template for PCR amplifications of the 16S-23S rRNA intergenic spacer (IGS) region with primers IGS1490’ (forward) and IGS132’ (reverse) (Laguerre et al., 1994). PCR amplification was carried out in a standard 30 μL reaction mixture including 1 μL of DNA template and 5 U of *Taq* DNA polymerase (Sangon Biotech (Shanghai) Co., Ltd.). Aliquots of amplified PCR products were visualized after electrophoresis in a 1.0% (w/v) agarose gel labeled with GoldView type I. Then, PCR products were digested separately with the endonucleases *HaeIII*, *MspI,* and *HhaI* (Laguerre et al., 1994) at 37°C for 10 h. The 16S-23S rDNA IGS type of each strain was designated after separation and visualization of restriction fragments by electrophoresis in 2.5% (w/v) agarose gel and UV-illumination.

### Phylogenetic identification of the isolates

**Figure 1:**
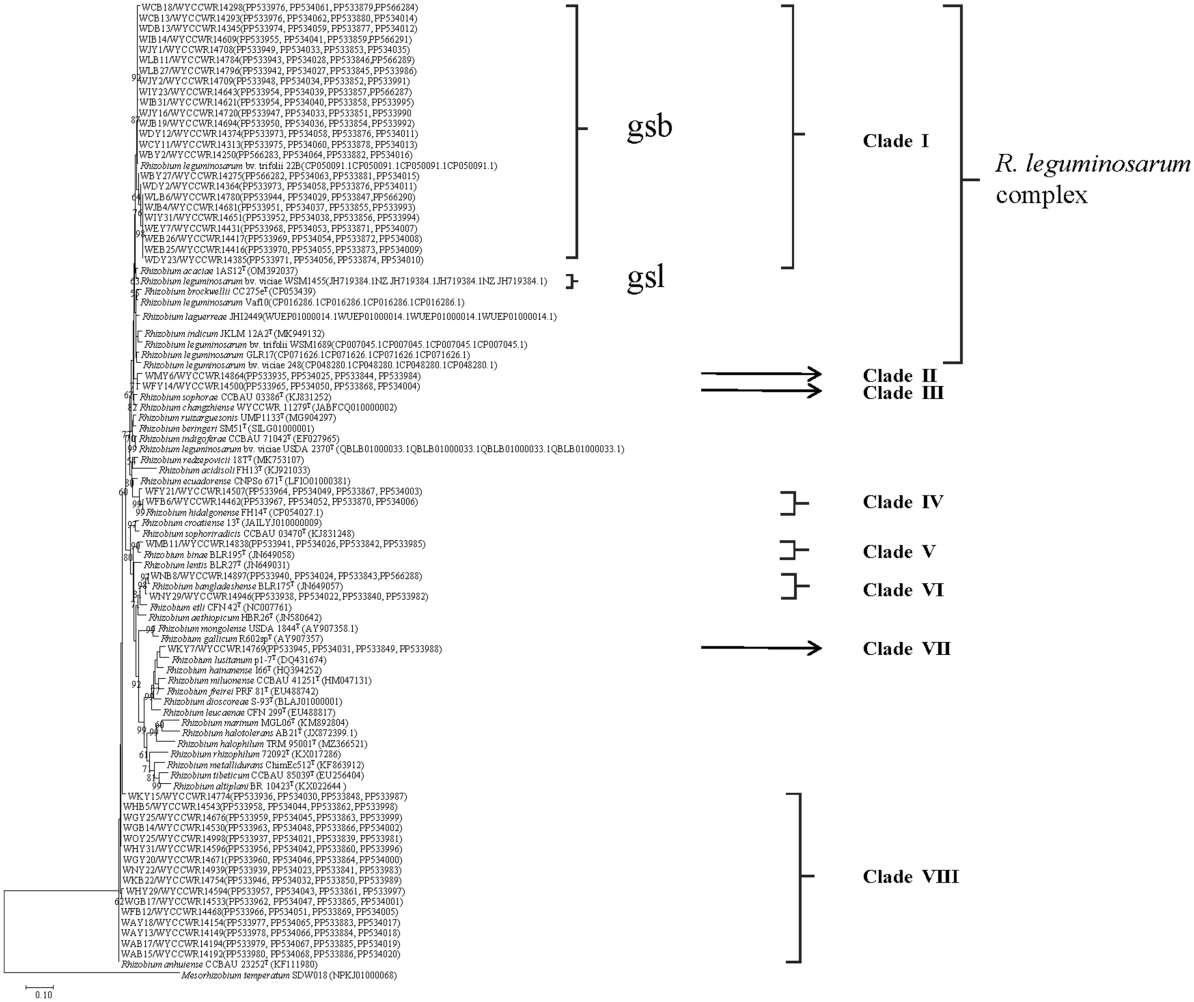
Maximum-likelihood phylogenetic tree based on concatenated *recA-atpD-dnaK-rpoB* gene sequences (1552 base pairs) showing the relationships of rhizobia isolated from studied *Pisum sativum* L. in Yunnan Province of China. The tree was constructed under the best-fit model (model GTR+G+I). Scale bar indicates 0.1 nucleotide substitution per site. Bootstrap confidence values (%) calculated for 500 replications >70% are indicated at the internodes.

Isolates sharing the same RFLP pattern of 16S-23S rDNA IGS in this study were designed as an IGS type and pick out 1–2 representative strains. The selected representative strains were amplified with forward primer P1 and reverse primer P6 to amplify the 16S rRNA gene (Laguerre et al., 1994). The PCR products were verified as mentioned above and were sent for commercial sequencing (Sangon Biotech (Shanghai) Co., Ltd.). The acquired sequences were compared in the NCBI database using the online BLASTN tool, and the sequences for type strains of defined *Rhizobium* species sharing similarities greater than 97.0% with the new isolates were extracted. The phylogenetic analysis was conducted in the MEGA 7.0 software (Kumar et al., 2016). Sequences were aligned using Clustal W, and the best model of sequence evolution was selected. Then, the phylogenetic tree was inferred by using the maximum-likelihood (ML) and the non-parametric bootstrap methods (500 pseudo-replications). DNA fragments of *recA* (coding for DNA recombination protein), *atpD* (encoding for ATP synthase beta chain), *dnak* (coding for DNA recombination protein), and *rpoB* (RNA Polymerase Beta Subunit) were amplified separately by PCR using the primer pairs *recA*41F/*recA*640R, *atpD*255F/*atpD*782R, *dnaK*1/*dnaK*4, and *rpoB*83F/*rpoB*1061R, respectively (Vinuesa et al., 2005; Zhang et al., 2012). PCR products verification, sequencing, and tree construction of each sequenced gene were performed as mentioned above. Furthermore, sequences of *atpD, recA, dnaK,* and *rpoB* genes were concatenated and aligned using Clustal W (Thompson et al., 1997). Distance calculation, construction of the concatenated housekeeping gene ML tree, and bootstrap analysis were performed in MEGA 7.0 as described above. The threshold of 97% similarity was used to defined the species of the isolates (Kumar et al., 2016). The sequences resulted from the alignment of the related genes have been deposited in the NCBI database (accession numbers are indicated on the trees, Figure 1; Supplementary Figures S1–S5).

### Phylogenetic analysis of symbiotic genes *nodC*

The symbiotic genes *nodC* (N-acetylglucosaminyl transferase) have been used as molecular markers to estimate the host specificity of rhizobia. These genes of the representative strains were amplified and forward sequenced by using the primer pair *nodC*540F/*nodC*1160R (Sarita et al., 2005) according to our previous report (Zhang et al., 2012). The obtained nucleotide sequences were deposited in the GenBank database. The phylogenetic analysis of these genes was performed as described before (Zhang et al., 2012).

### Correlation analysis between soil properties and rhizobial communities

A distance-based principal component analysis (PCA) was performed using CANOCO software version 5 to investigate the relationships among the soil properties (AP, AK, OM, AHN, Ec, and pH), other environmental factors (Alt, AveTmax, AveTmin, and AvePrecp), and the rhizobial community composition based on IGS genotypes.

### Nodulation and symbiotic efficiency test

The nodulation ability and symbiotic efficiency of the original host plants of the selected representative strains were tested. In brief, surface-sterilized pea seedlings were aseptically transferred in pots (1 plant/pot) containing sterile vermiculite as substrate and inoculated with 1 mL of rhizobial suspension (OD_600_ = 1.0). Plants were grown under greenhouse conditions and watered with sterile N-free nutrient solution as required (Zhang et al., 2012). Nodulation was evaluated 40 days after inoculation for all plants. Pea growth was estimated by weighting their dry root and shoot biomass and measuring leaf chlorophyll contents (SPAD chlorophyll meter). Uninoculated plants as a negative control were included, and all plant treatments were performed in three replicates. Data were analyzed by one-way ANOVA followed by an LSD post-hoc test (*p* = 0.001).

### Detection of physicochemical properties of representative strains

IAA production: As one of the phytohormones, IAA is involved in different plant growth and development processes such as the formation of the lateral roots and root hairs as well as increases the primary root length (Atrsaw et al., 2023). IAA production was common in plant-associated bacteria as part of a colonization strategy that involves phytostimulation and circumvention of plant defense mechanisms (Basbuga et al., 2021). In the present study, IAA production was determined for all the isolates (Nalini and Rao, 2014) in TY broth containing L-tryptophan (0.005 M) for 3 days of incubation at 28°C on a shaker at 180 rpm and then centrifuged at 3000 rpm for 20 min. Aliquot of 1 mL supernatant was mixed with 1 mL of Salkowski’s reagent. Uninoculated control was kept for comparison. The intensity of pink color developed within 30 min was measured at 530 nm in UV/VIS spectrophotometer. The quantity of IAA was determined by comparison with an IAA standard curve (Makarova et al., 2023). For acid and alkali resistance, rhizobial isolates that had similar growth patterns after 72 h on YMA plates in triplicate were selected, the pH of the medium was adjusted to 5, 6, 7, 8, 9, 10, and 11, and the control cultures were grown at YMA plates with pH7 (Zhongling et al., 2023).

#### Salt-tolerance

Rhizobial isolates that had similar growth patterns after 72 h on YMA plates in triplicate were selected, the NaCl concentration of the medium was adjusted to 0.01, 1, 2, 3, and 4% (w/v), and the control cultures were grown at YMA plates with 0.01% NaCl (Yoshikazu et al., 2021).

#### Drought-tolerance

PEG6000 was used to simulate drought conditions, rhizobial isolates were inoculated on YMA plates supplied with PEG6000 at the concentration of 0, 3, 5, 7, 10, and 15% (w/v), and the control cultures were grown at YMA plates with 0% PEG6000 (Saoussen et al., 2021; Liu-Qing et al., 2016).

#### Herbicide-tolerance

Rhizobial isolates were inoculated on YMA plates supplied with glyphosate (PMG) at concentration of 0, 0.6, 1.2, and 1.8% (w/v), and the control cultures were grown at YMA plates with 0% PMG (Philippe et al., 2021).

#### Temperature-tolerance

Rhizobial isolates were inoculated on YMA plates in triplicate and were incubated at 4°C, 10°C, 28°C, and 37°C. The pH of the medium was 7, and the control cultures were grown at 28°C. The strains that were able to grow at 37°C on YMA plates were further tested for growth at 45°C in TY broth for 10 min and then for 3 days at 28°C on a shaker at 180 rpm (Kulkarni and Nautiyal, 2000).

## Results

### Physicochemical characteristics of soils

All 15 sites differed from each other in their levels of organic matter, available nitrogen, available phosphorus, available potassium, pH, and conductivity (Supplementary Tables S1, S2). Field soil at YD-EY presented the highest contents of OM (62.8 g/kg soil) and AHN (388.87 g/kg soil), while the soil from site YN-SD contained the highest AP (138.93 mg/kg soil), soil from site YC-MD contained the highest AK (358.33 mg/kg soil), soil from site YC-YA contained the highest pH (7.74), and soil from site YC-DY contained the highest Ec (819.33 μs/cm soil). At the opposite, site YB-CN exhibited the lowest proportions of OM, site YC-SP exhibited the lowest proportions of available P in soil, site YC-DH exhibited the lowest proportions of available AHN in soil, and site YB-CN exhibited the lowest proportions of available K in soil. Eight of the sites had weakly alkaline soil above pH 7.16, while two of the sites had a neutral pH 7.02 and 6.93; the remaining site had weakly alkaline acidic.

### IGS PCR-RFLP analysis

In plant trapping tests, both the pea B and pea Y varieties formed nodules in all the soil samples. In total, 615 rhizobial isolates were obtained from the 15 sampling sites trapped with two different varieties of pea, which were distinguished into 33, 21 and 22 RFLP patterns with the restriction enzymes *HaeIII*, *MspI,* and *HhaI,* respectively. By combining all RFLP patterns, the IGS PCR-RFLP fingerprinting allowed the classification of all isolates into 43 IGS types (Table 2), with IGS type 1 representing the most abundant population (with 59 isolates), and types 42 and 43 representing the lowest abundant groups (1 isolates each) (Table 2). The most widely distributed IGS types were types 1 and 5 that were isolated from five sites, following by 4 types (2, 4, 6, 13) recovered from 4 sites, 5 types (7, 15, 18, 23, 30) occurred in 3 sites, 11 types were isolated from 2 sites, while the remaining 9 types were from only one site. In short, 20 IGS types were more competence on pea B than that on pea Y; the remaining 23 IGS types presented reverse situation. These results indicated that the richness and evenness of rhizobial IGS types varied across the sampling soil and pea varieties.

**Table 2:** Genetic groupings of *Rhizobium* isolates associated with *Pisum sativum* and their geographical distribution in the different sampling sites.

IGS RFLP Type	Isolate number	Representative isolate WYCCWR no./origin site^a^	MLSA similarity(%) with^b^								Isolates/ IGS type
			*Rac*	*Rch*	*Rin*	*Rhi*	*Rbi*	*Rba*	*Rha*	*Ran*	
22	10	WYCCWR14345/WDB13	98.4	96.8	97	93.4	92	90.8	88.5	95.3	*R. acaciae* (Clade I, C1)
25	9	WYCCWR14313/WCY11	98.4	96.8	97	93.4	92	90.8	88.5	95.3	
14	13	WYCCWR14364/WDY2	97.3	97.1	96.1	93.5	91.9	90.6	88.2	95.4	
8	21	WYCCWR14609/WIB14	98.4	96.8	97	93.4	92	90.8	88.5	95.3	
43	1	WYCCWR14643/WIY23	98.4	96.8	97	93.4	92	90.8	88.5	95.3	
2	50	WYCCWR14694/WJB19	98.4	96.8	97	93.4	92	90.8	88.5	95.3	
16	13	WYCCWR14708/WJY1	98.4	96.8	97	93.4	92	90.8	88.5	95.3	
2	50	WYCCWR14709/WJY2	98.4	96.8	97	93.4	92	90.8	88.5	95.3	
27	9	WYCCWR14720/WJY16	98.4	96.8	97	93.4	92	90.8	88.5	95.3	
12	15	WYCCWR14784/WLB11	98.4	96.8	97	93.4	92	90.8	88.5	95.3	
18	12	WYCCWR14796/WLB27	98.4	96.8	97	93.4	92	90.8	88.5	95.3	
19	11	WYCCWR14293/WCB13	98.4	96.8	97	93.4	92	90.8	88.5	95.3	
3	42	WYCCWR1462/WIB31	98.3	96.7	96.8	93.2	91.8	90.7	88.3	95.2	
29	8	WYCCWR14298/WCB18	97.4	95.7	96	92.5	90.8	90.2	87.4	94.3	
9	19	WYCCWR14250/WBY2	98.4	96.8	97	93.4	92	90.8	88.5	95.3	
38	4	WYCCWR14275/WBY27	97.8	97	96.7	93.6	91.8	90.5	88.2	95.1	
8	21	WYCCWR14374/WDY12	98.4	96.8	97	93.4	92	90.8	88.5	95.3	
41	2	WYCCWR14681/WJB4	97.3	96.6	96	93.2	92.1	90.8	88.6	95.3	
17	12	WYCCWR14780/WLB6	97.3	96.4	96	93.3	92.1	90.9	88.8	95.4	
5	34	WYCCWR14651/WIY31	97.3	96.6	96	93.2	92.1	90.8	88.6	95.3	
30	8	WYCCWR14431/WEY7	97.3	96.6	96	93.2	92.1	90.8	88.6	95.3	
40	3	WYCCWR14416/WEB25	97.3	96.6	96	93.2	92.1	90.8	88.6	95.3	
7	25	WYCCWR14417/WEB26	97.3	96.6	96	93.2	92.1	90.8	88.6	95.3	
7	25	WYCCWR14385/WDY23	97.3	96.6	96	93.2	92.1	90.8	88.6	95.3	
34	6	WYCCWR14864/WMY6	95.6	95.7	95.1	93.2	90.8	92.1	86.9	94.6	*R. genosp.* I (Clade II, C2)
23	10	WYCCWR14500/WFY14	96.3	96	96.9	93.2	90.8	90.1	87.5	94.9	*R. genosp*. II (Clade III, C3)
20	11	WYCCWR14462/WFB6	93.2	92.7	93.2	99.9	90.7	90.6	87.6	93.1	*R. hidalgonense* (Clade IV, C4)
35	6	WYCCWR14507/WFY21	94.7	93.5	94	97.9	90.3	90.2	87	93.3	
4	39	WYCCWR14838/WMB11	94.1	93.8	92.9	91.4	97.2	92.1	88.3	93.8	*R. binae* (Clade V, C5)
28	9	WYCCWR14897/WNB8	91.1	90.8	90.6	90.9	93.3	99.4	87.7	92.2	*R. bangladeshense* (Clade VI, C6)
13	14	WYCCWR14946/WNY29	91.4	91	90.4	90.6	93.6	97.9	87.5	93.5	
32	7	WYCCWR14769/WKY7	88.7	87.1	87.1	86.4	88.8	87.2	95.3	87.5	*R. genosp*. III (Clade VII, C7)
37	5	WYCCWR14774/WKY15	94	94	93.1	92.7	92.1	93.2	87.4	97.5	*R. anhuiense* (Clade VIII, C8)
39	4	WYCCWR14543/WHB5	94.9	94.6	93.9	93.3	92.7	92	87.8	99.2	
33	7	WYCCWR14676/WGY25	94.9	94.6	93.9	93.3	92.7	92	87.8	99.2	
21	11	WYCCWR14530/WGB14	94.9	94.6	93.9	93.3	92.7	92	87.8	99.2	
42	1	WYCCWR14596/WHY31	94.9	94.6	93.9	93.3	92.7	92	87.8	99.2	
36	5	WYCCWR14998/WOY25	94.9	94.6	93.9	93.3	92.7	92	87.8	99.2	
26	9	WYCCWR14594/WHY29	94.5	94.5	93.9	92.8	92.2	91.4	87.5	99.4	
11	16	WYCCWR14533/WGB17	94.7	94.5	93.8	92.7	92.2	91.5	87.4	98.9	
10	17	WYCCWR14754/WKB22	95	94.7	94	93.1	92.8	92.1	87.7	99.5	
24	10	WYCCWR14671/WGY20	94.9	94.6	93.9	93.2	92.7	92	87.6	99.4	
31	7	WYCCWR14939/WNY22	95	94.7	94	93.1	92.8	92.1	87.7	99.5	
15	13	WYCCWR14468/WFB12	94.9	94.6	94.1	93.2	92.8	92	87.9	100	
6	27	WYCCWR14154/WAY18	94.9	94.6	94.1	93.2	92.8	92	87.9	100	
1	59	WYCCWR14149/WAY13	94.9	94.6	94.1	93.2	92.8	92	87.9	100	
6	27	WYCCWR14192/WAB15	94.9	94.6	94.1	93.2	92.8	92	87.9	100	
1	59	WYCCWR14194/WAB17	94.9	94.6	94.1	93.2	92.8	92	87.9	100	
IGS type number:43	Total isolate number:615	Total representatives:48									Total species 8

^a^.Clusters or genomic species were defined by MLSA (*recA – atpD - dnaK-rpoB*).

^b^ MLSA similarity with the most related strains: Rac, R. acaciae 1AS12^T^; Rch, R. changzhiense WYCCWR 11279^T^; Rin, R. indicum JKLM 12A2^T^; Rhi, Rhizobium hidalgonense FH14^T^; Rbi, R. binae BLR195^T^; Rba, R.bangladeshense BLR175^T^; Rha, R.hainanense I66^T^; Ran, Rhizobium anhuiense CCBAU 23252^T^.

^c^.YC-MD, Mouding County, Chuxiong city; YC-YA, Yaoan county, Chuxiong city; YC-DY, Dayao county, Chuxiong city; YC-LF, Lufeng city, Chuxiong city; YC-DH, Donghua town, Chuxiong city; YC-WD, Wuding County, Chuxiong city; YC-SP, Shuangpai county, Chuxiong city; YC-NH, Nanhua county, Chuxiong city; YD-XY, Xiangyun county, Dali city; YD-WS, Weishan county, Dali city; YD-MD, Midu county, Dali city; YD-EY, Eryuan county, Dali city; YB-LY, Longyang district, Baoshan city; YB-CN, Changning county, Baoshan city; YB-SD, Shidian county, Baoshan city;

### Phylogenetic analysis of core genes and species affiliation of pea rhizobia

Nearly full-length 16S rRNA genes were successfully amplified and sequenced for 48 rhizobial isolates representing all the 43 IGS types and the 5 sites of origin (Table 2). All representative isolates were clustered together within the genus *Rhizobium,* and most of them presented 99.5–100% similarity in their 16S rRNA gene sequences with type strains for different defined *Rhizobium* species, including *R. trifolii* ATCC 14480^T^*, R. sophorae* CCBAU 03386^T^, *R. ruizarguesonis* UMP1133^T^, *R. leguminosarum* LMG 14904^T^ (USDA 2370^T^), *R. laguerreae FB206*^T^, *R. changzhiense* WYCCWR 11279^T^, *R. dioscoreae* strain S-93^T^, *R. hainanense* I66^T^, *R. miluonense* CCBAU 41251^T^, *R. paranaense* PRF 35^T^, and *R. sophoriradicis* CCBAU 03470^T^ (Supplementary Figure S1).

The representative isolates were divided into eight clades (C1–C8) in the phylogenetic tree based on their concatenated *recA*-*atpD*-*dnaK*-*rpoB* sequences (Figure 1). Twenty-four representative isolates from 21 IGS types (2, 3, 5, 7, 8, 9, 12, 14, 16, 17, 18, 19, 22, 25, 27, 29, 30, 38, 40, 41, and 43) covering 322 isolates (account for 52.5%) from 14 sampling sites were grouped in C1 cluster together with the type strain *R. acaciae* 1AS12^T^ at 97.3–98.4% similarities (Table 2). Thus, Cluster C1 was identified as *R. acaciae*. Cluster C2 contained *R. changzhiense* WYCCWR 11279^T^ and one representative isolates, covering 6 isolates (account for 0.98%) from 1 sampling sites, which shared 95.7% similarities with each other and less than 95.7% similarities with the other *Rhizobium* type strains; therefore, they were identified as *Rhizobium genosp.* I. Cluster C3 contained *R. indicum* JKLM 12A2^T^ and one representative isolates, covering 10 isolates (account for 1.62%) from 3 sampling sites, which shared 96.9% similarities with each other and less than 96.9% similarities with the other *Rhizobium* type strains; therefore, they were identified as *Rhizobium genosp.* II. Cluster C4 contained 2 IGS types (20 and 35) covering 17 isolates (account for 2.8%) from 2 sampling sites, and they showed 97.9–99.9% similarities with *Rhizobium hidalgonense* FH14^T^; so, this cluster was identified as *Rhizobium hidalgonense*. Cluster C5 contained IGS type 4 with 39 isolates (account for 6.3%) from 4 sampling sites that showed 97.2% similarities with *Rhizobium binae* BLR195^T^ and presented similarities less than 97% with the other *Rhizobium* species, so this cluster was identified as *R. binae*. Cluster C6 contained IGS types 13 and 28 with 23 isolates (account for 3.7%) from 5 sampling sites and showed 97.9–99.4% similarities with *Rhizobium bangladeshense* BLR175^T^ and presented similarities less than 97% with the other *Rhizobium* species, so this cluster was identified as *R. bangladeshense*. Cluster C7 contained IGS type 32 with 7 isolates (account for 1.1%) from the sampling site YC-YA and presented similarities less than 97% with the other *Rhizobium* species, so this cluster was identified as *Rhizobium genosp*. III. Cluster C8 contained IGS types 1, 6, 10, 11, 15, 21, 24, 26, 31, 33, 36, 37, 39, and 42 with 191 isolates (accounting for 30.7%) from 14 sampling sites, and they showed 97.5–100% similarities with *R. anhuiense* CCBAU 23252^T^ and less than 97% similarities with the other *Rhizobium* species; so, this cluster was identified as *R. anhuiense* (Table 2; Figure 1). The phylogenetic analyses of the single gene of *recA, atpD, dnaK,* and *rpoB* (Supplementary Figures S2–S5) were similar with that in the MLSA although some variations exist.

### Affinity of rhizobial groups with two cultivars of pea

In the present study, both pea varieties formed nodules in all the soil samples, and no obvious difference in the nodule numbers was observed between the pea varieties in each soil sample; while the nodule numbers in different sampling sites presented great variation (Supplementary Table S4). In general, the numbers of isolates from both pea varieties were different, with 306 and 309 isolates from pea B and pea Y, respectively. In most cases, strains belonging to the same IGS types were isolated from nodules of both peaB and peaY grown in the same soil samples. However, several IGS types presented different isolation frequencies on the two pea varieties and/or in different sampling sites (Supplementary Table S4). For example, types 29, 38, 40, and 41 were only isolated from peaB, while types 21, 28, 32, and 36 were only isolated from peaY plants. In addition, more type 1 and type 2 strains were isolated from peaB than that from peaY. In contrast, more strains of type 4 and type 6 were isolated from peaY than that from peaB. At the level of genomic species, C7 was only isolated from pea Y, while more C1, C3, C4, and C8 were also isolated from pea B than that from pea Y, with 164/158, 8/2, 11/6, and 102/89, respectively. In another hand, more *R. acaciae* and C2, C5, and C6 were isolated from pea Y than that from pea B, with ratio of 2/4, 13/6, and 6/17, respectively. Thus, rhizobial species seemed to have different responses to soil chemical characteristics and the environmental factors.

### Correlation analysis between rhizobial IGS types and soil properties

**Figure 2:**
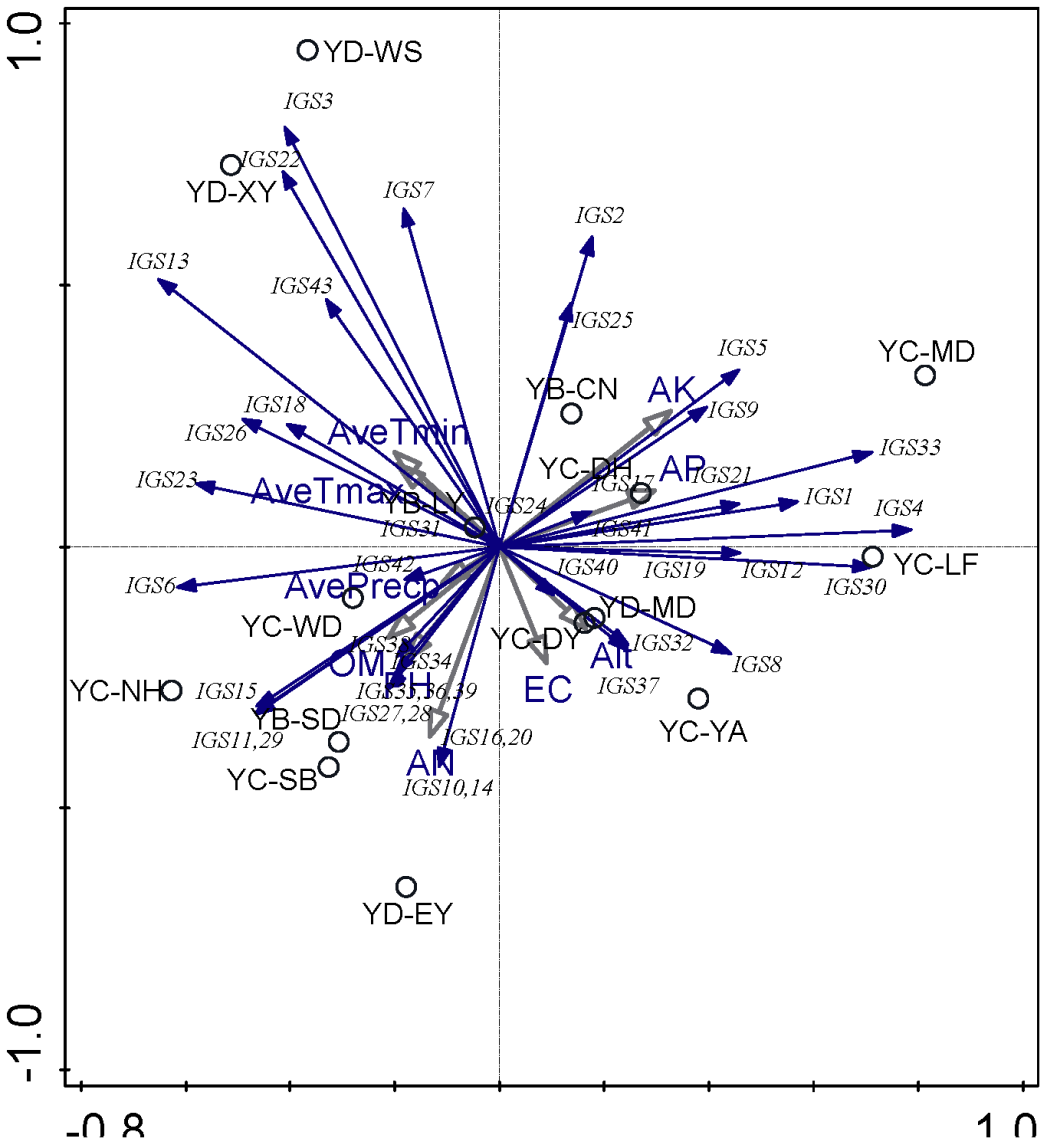
Principal component analysis (PCA) to relate the distribution of the 43 IGS types of isolates to physicochemical factors of soils collected from the different sites. Blue arrows indicate IGS types of rhizobia, grey arrows represent soil properties and circles represent sampling sites. The longer the arrow was, the greater the influence of the soil properties and the environmental factor presents on the distribution of the IGS types. The smaller the angle between the arrow and the IGS type was, the stronger the effect of the soil property on distribution of the IGS type.

PCA was used to explore the relationships between soil chemical properties and the rhizobial community composition based on IGS genotypes. According to PCA results (Figure 2), it seems that the soil factors presented greater effects on the distribution of rhizobial IGS types than the climate factors, while the nutrient factors (AN, AK, and AP) presented greater effects than the physical characters (pH and EC). Associations between some IGS types with certain sampling sites were revealed in this analysis. For example, i) IGS types 3, 7, and 22 encompassing 78 isolates were associated with sites YD-XY and YD-WS, and they were negatively correlated with EC and Alt values but positively related to average temperatures (Figure 2; Supplementary Table S1) that coincided with the soil properties of YD-XY and YD-WS (Table 1). Isolates of IGS type 13, 18, 23, and 26 were from five sampling sites (Table 2), and they presented relationships with the EC/Alt and AveTmin/AveTmax similar with IGS types 3, 7, and 22 but were the opposite of IGS types 8, 32, and 37 (Figure 2; Supplementary Table S1). iii) Isolates in 15 IGS types (6, 10, 11, 14, 15, 16, 20, 27, 28, 29, 34, 35, 36, 38, and 39) encompassing 150 isolates distributed across the seven sites (Table 2) were correlated with higher OM, AN, and pH but lower AK and AP, while those in another 15 IGS types (1, 4, 5, 9, 12, 17, 19, 21, 24, 30, 33, 40, and 41) presented opposite correlations (Figure 2). In general, the correlation between sampling sites and IGS types was not very clear, and the distribution of distinct rhizobial populations was influenced differently by edaphic factors.

At the genospecies level (Supplementary Figure S9), the climate factors of average temperatures presented effects on rhizobial distribution opposite of the other (soil) factors. *Rhizobium acaciae* was mainly distributed in Yao’an County, Muding County, Lufeng City, and Donghua Town of Chuxiong City. The distribution of *Rhizobium acaciae* was positively correlated with AK and Alt. *Rhizobium genosp*. I and *Rhizobium genosp*. II were mainly distributed in Wuding County, Chuxiong City, and its distribution was positively correlated with OM. *R. hidalgonense* was mainly distributed in Changning County, Baoshan City, and its distribution was positively correlated with EC, AN, AP, pH, and AvePrecp. *Rhizobium anhuiense* was mainly distributed in Muding County and Nanhua County of Chuxiong City, and its distribution was positively correlated with OM. *Rhizobium binae* was mainly distributed in Yao’an County, Chuxiong City, and its distribution was positively correlated with AK and Alt. *Rhizobium bangladeshense* was mainly distributed in Wuding County, Chuxiong City, and its distribution was positively correlated with OM. *Rhizobium genosp.* III was mainly distributed in Yao’an County, Chuxiong City, and its distribution was positively correlated with AK and Alt. This showed that different strains had strong competitive ability and could adapt to different soil environment and subsequent conditions.

### Phylogenetic analysis of *nodC*

**Figure 3:**
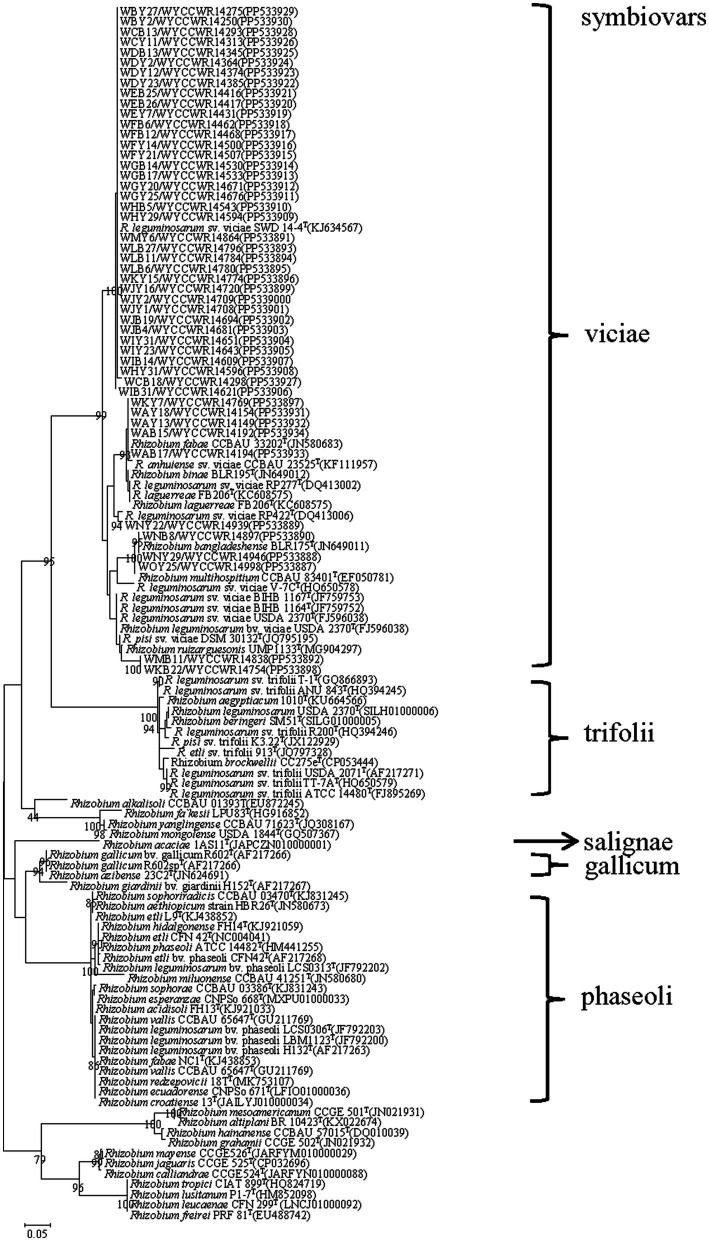
Maximum-likelihood phylogenetic tree based on symbiotic gene *nodC* (355 base pairs) showing the relationships of the rhizobia isolated from nodules of *Pisum sativum L*. in Yunnan Province of China. The tree was constructed using the maximum-likelihood method under the best-fit model (T92). Scale bar indicates 0.05 nucleotide substitutions per site. Bootstrap confidence values (%) calculated for 500 replications >70% are indicated at the internodes.

The *nodC* genes were amplified from all the 48 representative isolates. As shown in Figure 3, the *nodC* genes of all the representative isolates presented sequence similarities between 90.0 and 99.6, and all grouped with defined strains of symbiovar viciae (Figure 3).

### Symbiotic efficiency of rhizobial strains

**Figure 4:**
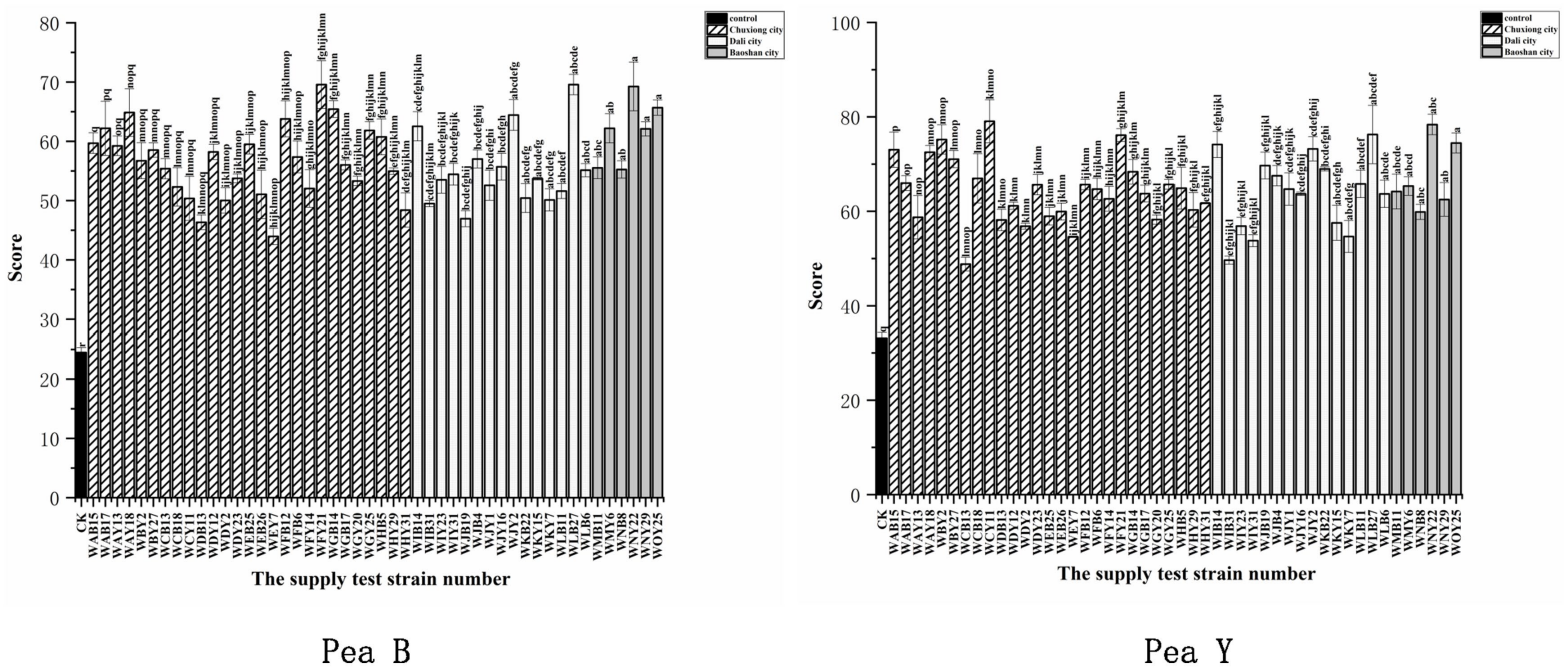
Analysis of the weighted mean for the determination of the symbiotic effects. Symbiotic performance of 48 representative strains evaluated on two varieties of pea grown under greenhouse conditions 40 days after inoculation, CK is the negative control (uninoculated plants). The experiment was conducted in triplicates. In addition, three subsamples were performed for each SPAD replication. Left for Pea B and right for Pea Y. Different letters above bars show significant differences (ANOVA + LSD test, *** *p* < 0.001). Bars indicate the standard error.

All the representative isolates for the 43 IGS types formed nodules on both pea B and peaY seedlings, with 20 to 90 nodules per plants (Supplementary Figure S6; Supplementary Table S3). Compared to the uninoculated control (*p* < 0.001), inoculation of all the representative isolates significantly increased leaf chlorophyll contents (33.7–88.8%) of pea B and (27.8–68.2%) pea Y plants (Supplementary Figure S7), and most of them significantly increased the total plant dry weight (51.6–207.4%) of pea B and (8.7–123%) of pea Y (Supplementary Figure S8), indicating that they were effective rhizobial symbionts. Among the tested isolates, four (WNY22, WOY25, WLB27, and WJY2) presented relatively greater promotion on both pea B and pea Y plants (154–207.3% and 95–123% increase, respectively, in relation to the control) than the others (Supplementary Table S3). Meanwhile, several isolates presented better promotion on nodulation or growth of plants with at least one of the two varieties, such as, WMY6, WAY18, WIB14, WCY11, WFY21, and WBY2 (Figure 4; Supplementary Figures S6–S8; Supplementary Table S3). In general, the high efficient strains formed approximately 50 nodules per plants on both varieties (Supplementary Table S3). Based on the symbiotic effects of representative strains, 15 symbiotic nitrogen fixing dominant strains were screened out from two pea varieties, among which 12 performed well in the symbiotic experiment on the two pea varieties, namely, WFY21, WAY18, WCB18, WLB27, WJY2, WIB14, WJB4, WNY22, WOY25, WMY6, WNY29, and WMB11 (Figure 4).

### Bio-physical and biochemical properties of representative strains

In determination of IAA production (Supplementary Table S3), the highest IAA producer (64.556 mg L^−1^) was WCB18, significantly higher than that of the other strains. In addition, three representative strains, namely, WCB13, WCY11, and WGB14, have relatively high levels of IAA production, indicating that some of the 615 isolates have the ability to produce IAA more effectively. As seen in the above study, the symbiotic effects of these four strains were also outstanding.

All the tested strains were unable to grow under pH5; 23 and 11 strains could not grow under pH6 and pH10, respectively; 13 representative strains, namely, WAB17, WBY2, WCB13, WCB18, WDY2, WDY23, WFB6, WHY29, WIY23, WKB22, WKY7, WMY5, and WNY29, could grow under pH11, while the other 35 strains could not. At a salt concentration of 1%, 9 strains, including WBY2, WBY27, WCB18, WFY14, WFY21, WGY20, WKY15, WLB27, and WNY29, could grow normally; at a salt concentration of 3%, six strains, namely, WBY2, WBY27, WCB18, WFY21, WFY14, and WGY20, could grow; at a salt concentration of 4%, five strains, namely, WBY2, WBY27, WCB18, WFY14, and WGY20, could grow, while the other 43 representative strains could not. All strains could not grow at 4° and 10°. Eight strains could grow at 37°C, namely, WAB17, WHY29, WJY2, WKB22, WKY7, WLB11, WLB27, and WNB8. Only three strains, namely, WHY29, WKB22, and WLB27, could grow under 45°C. Under simulated drought conditions with PEG6000 concentration of 3%, 7 strains could not grow, and the other 41 strains could. With 5% PEG6000, 12 strains could grow. With 7% PEG6000, most strains could not grow, but 5 strains, WAY13, WKB22, WKY15, WLB27, and WMB11, could. All strains could grow in medium supplied with different concentrations of herbicides (PMG).

## Discussion

Although pea plants have been cultured widely in different ecoregions in China, the diversity and geographic distribution of pea-nodulating rhizobia have rarely been studied (Ramirez et al., 2008). In the present study, we systematically investigated the diversity of *P. sativum* nodulating microsymbionts across 15 sampling sites within a tropical region of Yunnan Province (South-western China). Taking all the results of the 16S rRNA sequence analysis, the phylogeny of concatenated *recA-atpD-dnaK-rpoB* gene sequences, IGS PCR-RFLP analysis, and the *nodC* gene phylogeny, all the isolates obtained in this study were classified as a diverse community consisting of 43 IGS types within 8 genomic species belonging to the Rlc complex (Young et al., 2021) of the genus *Rhizobium*, and all of them belonged to the sv. viciae. These results provided some novel information on the diversity and geographic distribution of pea-nodulating rhizobia.

First, unique community composition of pea-nodulating rhizobial community was found in the studied subtropical region, which was dominated by *R. acaciae* (Hsouna et al., 2023) (52.5% in relative abundance) and *R. anhuiense* (30.7%) distributed across all the fifteen sampling sites. In addition, *Rhizobium genosp.* I*, Rhizobium genosp.* II, and *Rhizobium genosp.* III were identified as suspected new populations (Table 2). This community composition was different from those reported in other studies (Zhang et al., 2015; ChengYun et al., 2008; El Idrissi et al., 2020; El-Ghany et al., 2020). Especially, the similarity between C1 and *R. acaciae* 1AS12^T^ (Hsouna et al., 2023) isolated from *Acacia saligna* was 97.3–98.4% in the *recA-atpD-dnaK-rpoB* polytree. For more clarification, these strains were compared with the various species belonging to genospecies described previously by Young et al. (2021). The results showed that these strains were closely related to genospecies B(gsb) strain 22B, and the sequence similarity of concatenated gene sequences was 97–99.5%. In a previous study, the pea-nodulating rhizobia isolated from temperate region of China (Hebei Province), *R. sophorae,* and *R. indicum* were identified as dominant groups with relative abundances of 58 and 23%, respectively, while *R. changzhiense*, *R. anhuiense,* and a novel *Rhizobium* genospecies were the minor groups (Zhang et al., 2019). Another study revealed that *R. leguminosarum* and *R. etli* were the pea-nodulating rhizobia in several sites of subtropical region in China (ChengYun et al., 2008). Similar studies for isolates from other countries evidenced that pea-nodulating rhizobia were *R. leguminosarum* in Egypt (Yanni and Dazzo, 2010) and India (Stella et al., 2021) and *R. acidisoli* in Morocco (El Idrissi et al., 2020), while *Rhizobium laguerreae, R. ruizarguesonis*, and two putative genospecies, dominated by *R. laguerreae* (63%), were the pea-nodulating rhizobia in Tunisia (Ilahi et al., 2021). Considering the results in the present study and those mentioned above, it could be estimated that pea plants have selected rhizobia adapted to the local conditions in different geographic regions and biogeographic patterns exist for pea-associated.

Second, except the three dominant species, the two minor species *R. binae* and *R. bangladeshense* were not reported for pea-nodulating rhizobia previously. Among them, sv. viciae was a novel symbiovar in *R. hainanense* since strains belonging to sv. viciae have been reported in *R. hidalgonense* (Efstathiadou et al., 2020), *R. bangladeshense,* and *R. binae* (Maluk et al., 2022) for rhizobial isolates from *Vicia, Lathyrus,* and *Lens*. In our present study, the detection of similar but not identical *nodC* genes among the isolates evidenced that the *nodC* genes in pea-nodulating rhizobia had the same origin but they have diversified in association with different species. In addition, the dominant population in this study was previously reported as symbiovar salignae (Hsouna et al., 2022), but in this study it belongs to symbiovar viciae, which were similar to that found in soybean rhizobia (Man et al., 2008) and common bean rhizobia (Zhang et al., 2015; Li et al., 2016), and lateral transfer of symbiotic genes may have occurred. Together with the results of previous studies, our findings in the present study evidenced that the pea plant has strong selection for its microsymbionts both the chromosome background (only the *R. leguminosarum* complex) and the nodulation gene background (sv. viciae), although it has been cultured worldwide under different conditions. To the best of our knowledge, this is the first report that *R. acaciae* strains belonged to sv. viciae. Symbiovar *viciae* is widely distributed around the world and has strong heterogeneity. In the study of genetic diversity and phylogeny of indigenous rhizobia nodulating faba bean (*Vicia faba* L.) in Greece (Efstathiadou et al., 2020), this is the first report that *R. hidalgonense* strains belonged to sv. viciae, and it was confirmed that sv. viciae involves strains with highly diverse *nodC* genes, independent of chromosomal background (Álvarez-Martínez et al., 2009; Kumar et al., 2015). Villadas et al. (2017), Belhadi et al. (2018), Zhang et al. (2022), and Ampomah and Huss-Danell (2016) separately reported on southern Spain, Bejaia region of Algeria, North China, Mesoamerica and the Middle East and Sweden different populations of *Rhizobium* belong sv. viciae. Shamseldin et al. (2023) first report that *R. azibense* strains belonged to sv. viciae, and this finding showed the existence of symbiotic gene horizontal transfer events during the coevolution of *R. azibense* with *P. vulgaris* and *V. faba* in their respective distribution centers of Mesoamerica and the Middle East.

Third, the correlation analysis among the sampling sites, the environmental factors, and the distribution of rhizobial populations (Figure 2) gave us some interesting information. i) No clear association between sampling sites and rhizobial populations were detected in this study, which was different from the cases of soybean (Zhang et al., 2011) and common bean (Verástegui-Valdés et al., 2014). This difference might be related to the fact that the variation of environmental factors among the sampling area (sites) was not so apparent as that in the previous studies because the pH values and/or the salinity (Ec) values of the soils involved in the three mentioned studies were quite different. ii) Greater effects of soil characters than that of climate on the distribution of pea-associated rhizobial populations were observed in this study, which might be explained by the fact that rhizobia are soil-borne bacteria. Meanwhile, AP, AK, and AN presented greater effects than the other factors, which was similar to the previous reports (Zhang et al., 2011). iii) Another interesting point is that average temperatures were described as an important factor affecting the distribution of rhizobia. Three pea-nodulating rhizobial strains maintained no growth at 5 and 10°C but enhanced along the increase of temperature from 15°C to 30°C; correspondingly, no nodulation was found at temperature lower than 15°C, and the best nodulation occurred at 20°C (Serova et al., 2024). In addition, pea nodulation was adversely affected at 30°C. These results could explain why temperature could be a determinant for rhizobial distribution.

Fourth, our results also imply the necessity to screen out high-quality rhizobial strains with strong adaptability to local conditions to produce inoculants since the improvement of growth and yield of pea plant varied significantly among the isolates (Figure 4). These effects might be directly related to the capacity of N_2_ fixation of the isolates (Kristina and Abdollah, 2019), plant growth promoting traits (Twinkle et al., 2021), and their adaptation ability to the local factors (Jia et al., 2013). The results of nodulation tests (Figure 4) demonstrated that i) the nodule number induced on the host by inoculation was not a suitable criterion for selecting the efficient rhizobia. In this study, the four most efficient strains (WLB27, WJY2, WNY22, and WOY25) induced approximately 50 nodules per plants, and strains induced less (Gurkanli, 2021) nodules presented relatively low promotion on pea plant growth (Supplementary Table S4), while more nodules (80–90 per plants) did not increase the plant growth promotion. This observation was similar with that reported by He et al. (2011). In addition, the PGP effects of rhizobial strains may be related to their multiple traits, not a single trait, since the four most effective strains only present low IAA production (2.3–6.0 mg/L) (Supplementary Table S5), while WCB18 was the best IAA producer (64.6 mg/L), but not the best PGP strain.

In conclusion, our study demonstrated the existence of unique rhizobial community associated with pea cultivars in Yunnan Province, composing of *R. acaciae*, *R. anhuiense*, *R. binae, R. bangladeshense, R. hidalgonense,* and three suspected unknown species (*Rhizobium* genosp. I–III), with *R. acaciae* (52.5%) and *R. anhuiense* (30.7) as the dominant species. Moreover, *R. acaciae* and *R. anhuiense* occurred in 15 tested soil types, indicating that strains of these species could be better competitors and adapt to different soil conditions. Finally, all strains belonged to the sv. viciae, regardless of their species affiliation. The genetic diversity of pea-associated rhizobia from Yunnan Province provided a significant guideline for local pea cultivation and breeding efforts, as well as for the site-specific selection of efficient rhizobial genetic types.

## Data Availability

The datasets presented in this study can be found in online repositories. The names of the repository and accession numbers can be found in the article/Supplementary material.
