# Supplementary material for: Rhizobiumacaciae and R. anhuiense are the dominant rhizobial symbionts of Pisum sativum L. from Yunnan-Guizhou Plateau
Source: Front Microbiol. 2024 Sep 26;15:1437586. doi: 10.3389/fmicb.2024.1437586 (PMC11464311; doi:10.3389/fmicb.2024.1437586)
Supplement: Supplementary file 1 [file Presentation_1.PPTX]

## Slide 1
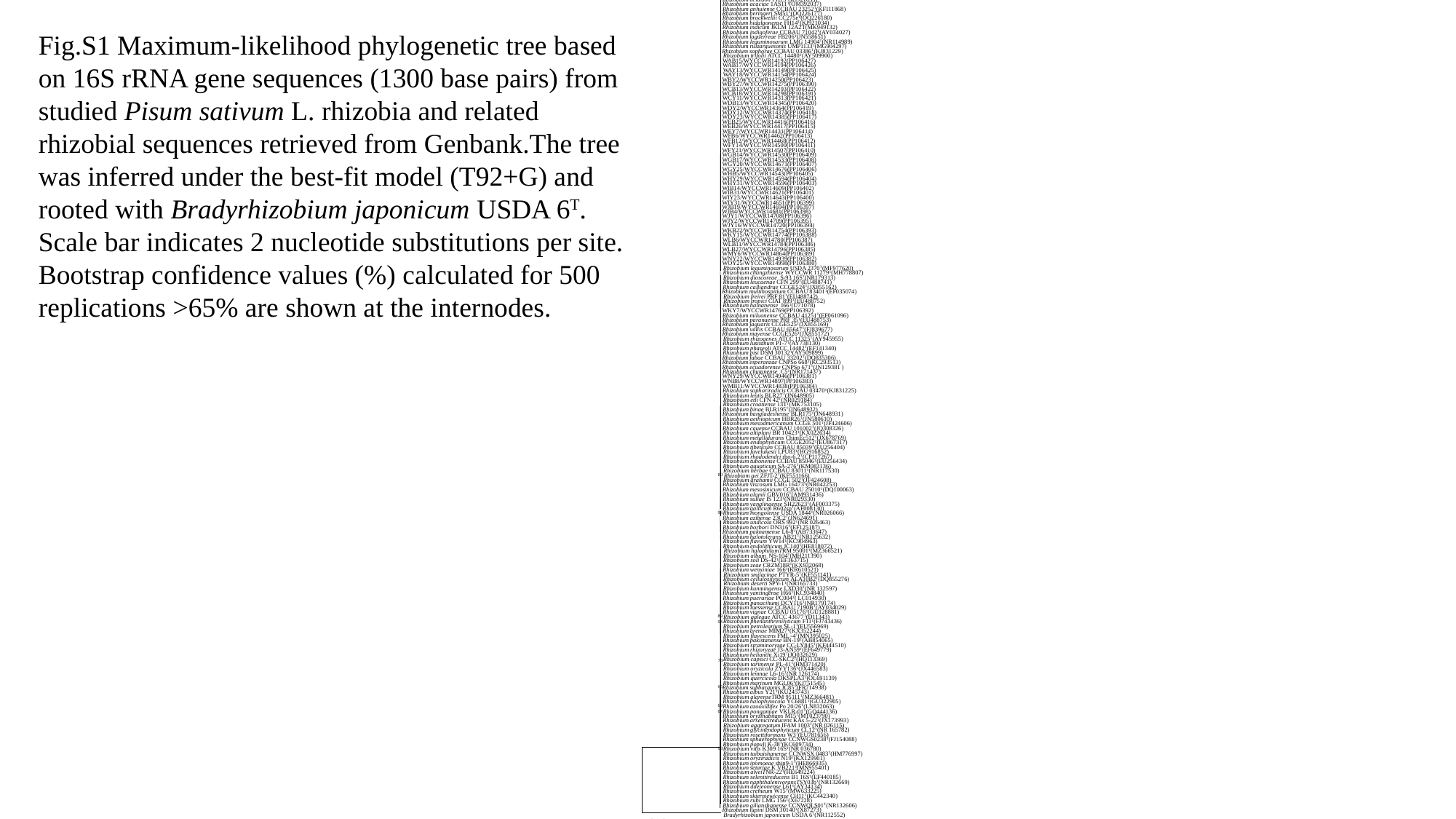

Rhizobium acidisoli FH13T(KJ921033)
 Rhizobium acaciae 1AS11T(OM392037)
 Rhizobium anhuiense CCBAU 23252T(KF111868)
 Rhizobium beringeri SM51T(OQ226177)
 Rhizobium brockwellii CC275eT(OQ226180)
 Rhizobium hidalgonense FH14T(KJ921034)
 Rhizobium indicum JKLM 12A2T(MK949132)
 Rhizobium indigoferae CCBAU 71042T(AY034027)
 Rhizobium laguerreae FB206T(JN558651)
 Rhizobium leguminosarum LMG 14904T(NR114989)
 Rhizobium ruizarguesonis UMP1133T(MG904297)
 Rhizobium sophorae CCBAU 03386T(KJ831229)
 Rhizobium trifolii ATCC 14480T(AY509900)
 WAB15/WYCCWR14192(PP106427)
 WAB17/WYCCWR14194(PP106426)
 WAY13/WYCCWR14149(PP106425)
 WAY18/WYCCWR14154(PP106424)
 WBY2/WYCCWR14250(PP106423)
 WBY27/WYCCWR14275(PP106390)
 WCB13/WYCCWR14293(PP106422)
 WCB18/WYCCWR14298(PP106391)
 WCY11/WYCCWR14313(PP106421)
 WDB13/WYCCWR14345(PP106420)
 WDY2/WYCCWR14364(PP106419)
 WDY12/WYCCWR14374(PP106418)
 WDY23/WYCCWR14385(PP106417)
 WEB25/WYCCWR14416(PP106416)
 WEB26/WYCCWR14417(PP106415)
 WEY7/WYCCWR14431(PP106414)
 WFB6/WYCCWR14462(PP106413)
 WFB12/WYCCWR14468(PP106412)
 WFY14/WYCCWR14500(PP106411)
 WFY21/WYCCWR14507(PP106410)
 WGB14/WYCCWR14530(PP106409)
 WGB17/WYCCWR14533(PP106408)
 WGY20/WYCCWR14671(PP106407)
 WGY25/WYCCWR14676(PP106406)
 WHB5/WYCCWR14543(PP106405)
 WHY29/WYCCWR14594(PP106404)
 WHY31/WYCCWR14596(PP106403)
 WIB14/WYCCWR14609(PP106402)
 WIB31/WYCCWR14621(PP106401)
 WIY23/WYCCWR14643(PP106400)
 WIY31/WYCCWR14651(PP106399)
 WJB19/WYCCWR14694(PP106397)
 WJB4/WYCCWR14681(PP106398)
 WJY1/WYCCWR14708(PP106396)
 WJY2/WYCCWR14709(PP106395)
 WJY16/WYCCWR14720(PP106394)
 WKB22/WYCCWR14754(PP106393)
 WKY15/WYCCWR14774(PP106388)
 WLB6/WYCCWR14780(PP106387)
 WLB11/WYCCWR14784(PP106386)
 WLB27/WYCCWR14796(PP106385)
 WMY6/WYCCWR14864(PP106389)
 WNY22/WYCCWR14939(PP106382)
 WOY25/WYCCWR14998(PP106380)
 Rhizobium leguminosarum USDA 2370T(MF977620)
 Rhizobium changzhiense WYCCWR 11279T(MH778807)
 Rhizobium dioscoreae S-93 16ST(NR179313)
 Rhizobium leucaenae CFN 299T(EU488741)
 Rhizobium calliandrae CCGE524T(JX855162)
 Rhizobium multihospitium CCBAU 83401T(EF035074)
 Rhizobium freirei PRF 81T(EU488742)
 Rhizobium tropici CIAT 899T(EU488752)
 Rhizobium hainanense I66T(U71078)
 WKY7/WYCCWR14769(PP106392)
 Rhizobium miluonense CCBAU 41251T(EF061096)
 Rhizobium paranaense PRF 35T(EU488753)
 Rhizobium jaguaris CCGE525T(JX855169)
 Rhizobium vallis CCBAU 65647T(FJ839677)
 Rhizobium mayense CCGE526T(JX855172)
 Rhizobium rhizogenes ATCC 11325T(AY945955)
 Rhizobium lusitanum P1-7T(AY738130)
 Rhizobium phaseoli ATCC 14482T(EF141340)
 Rhizobium pisi DSM 30132T(AY509899)
 Rhizobium fabae CCBAU 33202T(DQ835306)
 Rhizobium esperanzae CNPSo 668T(KC293513)
 Rhizobium ecuadorense CNPSo 671T(JN129381 )
 Rhizobium chutanense C5T(NR171437)
 WNY29/WYCCWR14946(PP106381)
 WNB8/WYCCWR14897(PP106383)
 WMB11/WYCCWR14838(PP106384)
 Rhizobium sophoriradicis CCBAU 03470T(KJ831225)
 Rhizobium lentis BLR27T(JN648905)
 Rhizobium etli CFN 42T(NR029184)
 Rhizobium croatiense 13TT(MK753105)
 Rhizobium binae BLR195T(JN648932)
 Rhizobium bangladeshense BLR175T(JN648931)
 Rhizobium aethiopicum HBR26T(JN580610)
 Rhizobium mesoamericanum CCGE 501T(JF424606)
 Rhizobium cauense CCBAU 101002T(JQ308326)
 Rhizobium altiplani BR 10423T(KX022634)
 Rhizobium metallidurans ChimEc512T(JX678769)
 Rhizobium endophyticum CCGE2052T(EU867317)
 Rhizobium tibeticum CCBAU 85039T(EU256404)
Fig.S1 Maximum-likelihood phylogenetic tree based on 16S rRNA gene sequences (1300 base pairs) from studied Pisum sativum L. rhizobia and related rhizobial sequences retrieved from Genbank.The tree was inferred under the best-fit model (T92+G) and rooted with Bradyrhizobium japonicum USDA 6T. Scale bar indicates 2 nucleotide substitutions per site. Bootstrap confidence values (%) calculated for 500 replications >65% are shown at the internodes.
 Rhizobium favelukesii LPU83T(HG916852)
 Rhizobium rhododendri rho-6.2T(CP117267)
 Rhizobium tubonense CCBAU 85046T(EU256434)
 Rhizobium aquaticum SA-276T(KM083136)
 Rhizobium herbae CCBAU 83011T(NR117530)
 Rhizobium gei ZFJT-2T(KF551166)
 Rhizobium grahamii CCGE 502T(JF424608)
 Rhizobium viscosum LMG 16473T(NR042253)
 Rhizobium mesosinicum CCBAU 25010T(DQ100063)
 Rhizobium alamii GBV016T(AM931436)
 Rhizobium sullae IS 123T(NR029330)
 Rhizobium yanglingense SH22623T(AF003375)
 Rhizobium gallicum R602spT(AF008130)
 Rhizobium mongolense USDA 1844T(NR026066)
 Rhizobium azibense 23C2T(JN624691)
 Rhizobium undicola ORS 992T(NR 026463)
 Rhizobium borbori DN316T(EF125187)
 Rhizobium paknamense L6-8T(AB733647)
 Rhizobium halotolerans AB21T(NR125632)
 Rhizobium flavum YW14T(KC904963)
 Rhizobium endolithicum JC140T(HE818072)
 Rhizobium halophilumTRM 95001T(MZ366521)
 Rhizobium album NS-104T(MH211390)
 Rhizobium soli DS-42T(EF363715)
 Rhizobium zeae CRZM18RT(KX932068)
 Rhizobium wenxiniae 166T(KR610521)
 Rhizobium smilacinae PTYR-5T(KF551141)
 Rhizobium cellulosilyticum ALA10B2T(DQ855276)
 Rhizobium deserti SPY-1T(NR165733)
 Rhizobium kunmingense LXD30T(NR 132597)
 Rhizobium yantingense H66T(KC934840)
 Rhizobium puerariae PC004T( LC014930)
 Rhizobium panacihumi DCY116T(NR179174)
 Rhizobium loessense CCBAU 7190BT(AY034029)
 Rhizobium vignae CCBAU 05176T(GU128881)
 Rhizobium galegae ATCC 43677T(D11343)
 Rhizobium phenanthrenilyticum F11T(FJ743436)
 Rhizobium petrolearium SL-1T(EU556969)
 Rhizobium arenae MIM27T(KX352244)
 Rhizobium flavescens FML -4T(MN395025)
 Rhizobium pakistanense BN-19T(AB854065)
 Rhizobium straminoryzae CC-LY845T(KF444510)
 Rhizobium rhizoryzae J3-AN59T(EF649779)
 Rhizobium helianthi Xi19T(JQ032629)
 Rhizobium capsici CC-SKC2T(HQ113369)
 Rhizobium tarimense PL-41T(HM371420)
 Rhizobium oryzicola ZYY136T(JX446583)
 Rhizobium lemnae L6-16T(NR 126174)
 Rhizobium quercicola DKSPLA3T(OL691139)
 Rhizobium marinum MGL06T(KJ751545)
 Rhizobium subbaraonis JC85T(FR714938)
 Rhizobium albus Y21T(KU245743)
 Rhizobium alarenseTRM 95111T(MZ366481)
 Rhizobium halophytocola YC6881T(GU322905)
 Rhizobium azooxidifex Po 20/26T(LN832063)
 Rhizobium pongamiae VKLR-01T(GQ444136)
 Rhizobium oryzihabitans M15T(MT023790)
 Rhizobium arsenicireducens KAs 5-22T(JX173993)
 Rhizobium aggregatum IFAM 1003T(NR 026115)
 Rhizobium glycinendophyticum CL12T(NR 165782)
 Rhizobium rosettiformans W3T(EU781656)
 Rhizobium sphaerophysae CCNWGS0238T(FJ154088)
 Rhizobium populi K-38T(KC609734)
 Rhizobium vitis K309 16ST(NR 036780)
 Rhizobium taibaishanense CCNWSX 0483T(HM776997)
 Rhizobium oryziradicis N19T(KX129901)
 Rhizobium ipomoeae shin9-1T(HE866935)
 Rhizobium setariae K VB221T(MN955401)
 Rhizobium alveiTNR-22T(HE649224)
 Rhizobium selenitireducens B1 16ST(EF440185)
 Rhizobium naphthalenivoransTSY03bT(NR132669)
 Rhizobium daejeonense L61T(AY34134)
80
96
89
93
73
65
69
67
98
 Rhizobium cremeum W15T(MW633225)
 Rhizobium skierniewicense CH11T(KC442340)
 Rhizobium rubi LMG 156T(X67228)
 Rhizobium qilianshanense CCNWQLS01T(NR132606)
 Rhizobium lupini DSM 30140T(X87273)
 Bradyrhizobium japonicum USDA 6T(NR112552)
2

## Slide 2
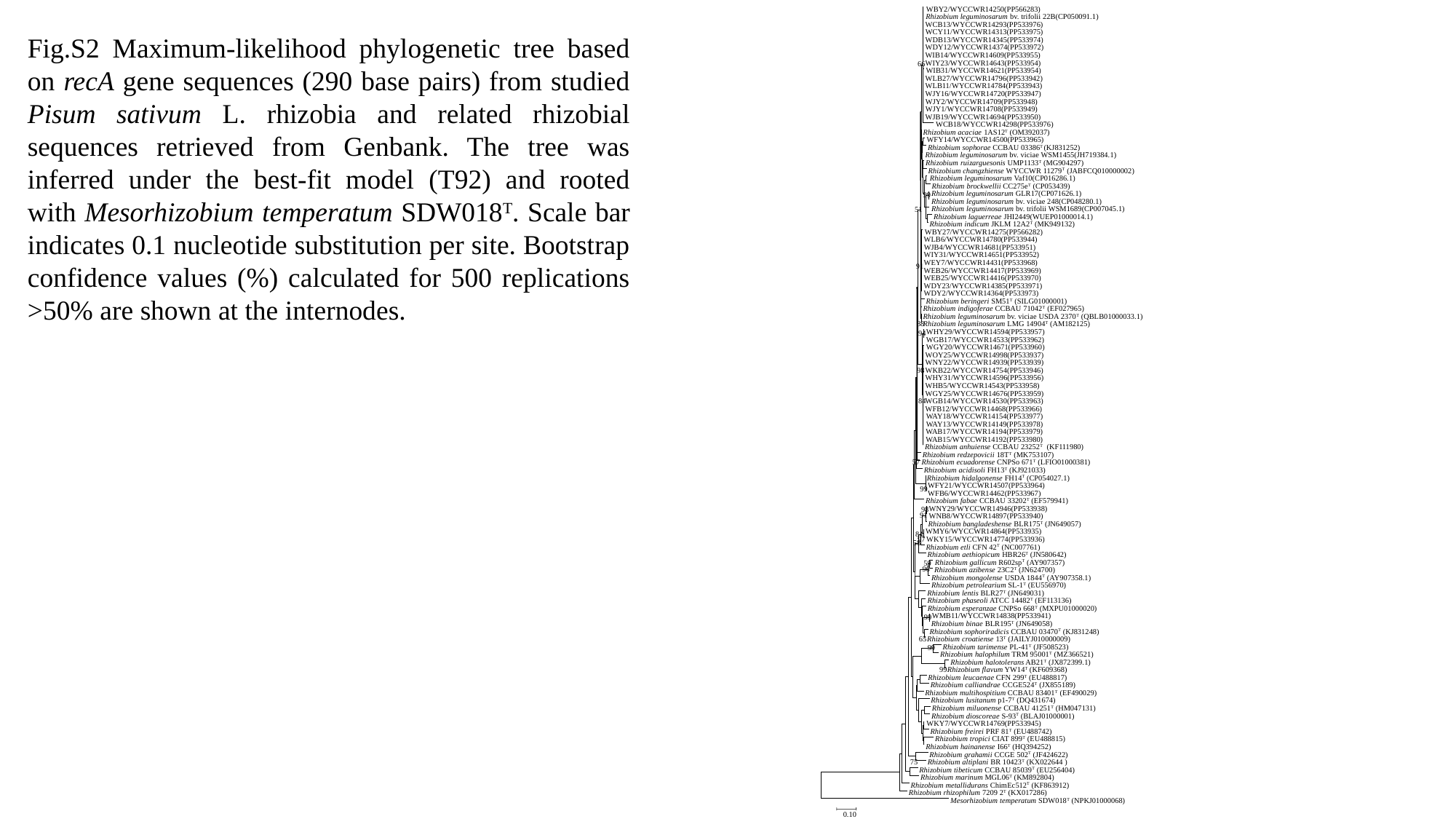

WBY2/WYCCWR14250(PP566283)
 Rhizobium leguminosarum bv. trifolii 22B(CP050091.1)
 WCB13/WYCCWR14293(PP533976)
 WCY11/WYCCWR14313(PP533975)
 WDB13/WYCCWR14345(PP533974)
 WDY12/WYCCWR14374(PP533972)
 WIB14/WYCCWR14609(PP533955)
 WIY23/WYCCWR14643(PP533954)
 WIB31/WYCCWR14621(PP533954)
 WLB27/WYCCWR14796(PP533942)
 WLB11/WYCCWR14784(PP533943)
 WJY16/WYCCWR14720(PP533947)
 WJY2/WYCCWR14709(PP533948)
 WJY1/WYCCWR14708(PP533949)
 WJB19/WYCCWR14694(PP533950)
 WCB18/WYCCWR14298(PP533976)
 Rhizobium acaciae 1AS12T (OM392037)
 WFY14/WYCCWR14500(PP533965)
 Rhizobium sophorae CCBAU 03386T (KJ831252)
 Rhizobium leguminosarum bv. viciae WSM1455(JH719384.1)
 Rhizobium ruizarguesonis UMP1133T (MG904297)
 Rhizobium changzhiense WYCCWR 11279T (JABFCQ010000002)
 Rhizobium leguminosarum Vaf10(CP016286.1)
 Rhizobium brockwellii CC275eT (CP053439)
 Rhizobium leguminosarum GLR17(CP071626.1)
 Rhizobium leguminosarum bv. viciae 248(CP048280.1)
 Rhizobium leguminosarum bv. trifolii WSM1689(CP007045.1)
 Rhizobium laguerreae JHI2449(WUEP01000014.1)
 Rhizobium indicum JKLM 12A2T (MK949132)
 WBY27/WYCCWR14275(PP566282)
 WLB6/WYCCWR14780(PP533944)
 WJB4/WYCCWR14681(PP533951)
 WIY31/WYCCWR14651(PP533952)
 WEY7/WYCCWR14431(PP533968)
 WEB26/WYCCWR14417(PP533969)
 WEB25/WYCCWR14416(PP533970)
 WDY23/WYCCWR14385(PP533971)
 WDY2/WYCCWR14364(PP533973)
 Rhizobium beringeri SM51T (SILG01000001)
 Rhizobium indigoferae CCBAU 71042T (EF027965)
 Rhizobium leguminosarum bv. viciae USDA 2370T (QBLB01000033.1)
 Rhizobium leguminosarum LMG 14904T (AM182125)
 WHY29/WYCCWR14594(PP533957)
 WGB17/WYCCWR14533(PP533962)
 WGY20/WYCCWR14671(PP533960)
 WOY25/WYCCWR14998(PP533937)
 WNY22/WYCCWR14939(PP533939)
 WKB22/WYCCWR14754(PP533946)
 WHY31/WYCCWR14596(PP533956)
 WHB5/WYCCWR14543(PP533958)
 WGY25/WYCCWR14676(PP533959)
 WGB14/WYCCWR14530(PP533963)
 WFB12/WYCCWR14468(PP533966)
 WAY18/WYCCWR14154(PP533977)
 WAY13/WYCCWR14149(PP533978)
 WAB17/WYCCWR14194(PP533979)
 WAB15/WYCCWR14192(PP533980)
 Rhizobium anhuiense CCBAU 23252T (KF111980)
 Rhizobium redzepovicii 18TT (MK753107)
 Rhizobium ecuadorense CNPSo 671T (LFIO01000381)
 Rhizobium acidisoli FH13T (KJ921033)
 Rhizobium hidalgonense FH14T (CP054027.1)
 WFY21/WYCCWR14507(PP533964)
 WFB6/WYCCWR14462(PP533967)
 Rhizobium fabae CCBAU 33202T (EF579941)
 WNY29/WYCCWR14946(PP533938)
 WNB8/WYCCWR14897(PP533940)
 Rhizobium bangladeshense BLR175T (JN649057)
 WMY6/WYCCWR14864(PP533935)
 WKY15/WYCCWR14774(PP533936)
 Rhizobium etli CFN 42T (NC007761)
 Rhizobium aethiopicum HBR26T (JN580642)
 Rhizobium gallicum R602spT (AY907357)
 Rhizobium azibense 23C2T (JN624700)
 Rhizobium mongolense USDA 1844T (AY907358.1)
 Rhizobium petrolearium SL-1T (EU556970)
 Rhizobium lentis BLR27T (JN649031)
 Rhizobium phaseoli ATCC 14482T (EF113136)
 Rhizobium esperanzae CNPSo 668T (MXPU01000020)
66
99
51
91
88
94
98
84
57
99
98
91
84
97
58
59
96
 WMB11/WYCCWR14838(PP533941)
99
 Rhizobium binae BLR195T (JN649058)
 Rhizobium sophoriradicis CCBAU 03470T (KJ831248)
65
 Rhizobium croatiense 13T (JAILYJ010000009)
 Rhizobium tarimense PL-41T (JF508523)
99
 Rhizobium halophilum TRM 95001T (MZ366521)
 Rhizobium halotolerans AB21T (JX872399.1)
99
 Rhizobium flavum YW14T (KF609368)
 Rhizobium leucaenae CFN 299T (EU488817)
 Rhizobium calliandrae CCGE524T (JX855189)
 Rhizobium multihospitium CCBAU 83401T (EF490029)
 Rhizobium lusitanum p1-7T (DQ431674)
 Rhizobium miluonense CCBAU 41251T (HM047131)
 Rhizobium dioscoreae S-93T (BLAJ01000001)
 WKY7/WYCCWR14769(PP533945)
 Rhizobium freirei PRF 81T (EU488742)
 Rhizobium tropici CIAT 899T (EU488815)
 Rhizobium hainanense I66T (HQ394252)
 Rhizobium grahamii CCGE 502T (JF424622)
75
 Rhizobium altiplani BR 10423T (KX022644 )
 Rhizobium tibeticum CCBAU 85039T (EU256404)
 Rhizobium marinum MGL06T (KM892804)
 Rhizobium metallidurans ChimEc512T (KF863912)
 Rhizobium rhizophilum 7209 2T (KX017286)
 Mesorhizobium temperatum SDW018T (NPKJ01000068)
0.10
Fig.S2 Maximum-likelihood phylogenetic tree based on recA gene sequences (290 base pairs) from studied Pisum sativum L. rhizobia and related rhizobial sequences retrieved from Genbank. The tree was inferred under the best-fit model (T92) and rooted with Mesorhizobium temperatum SDW018T. Scale bar indicates 0.1 nucleotide substitution per site. Bootstrap confidence values (%) calculated for 500 replications >50% are shown at the internodes.

## Slide 3
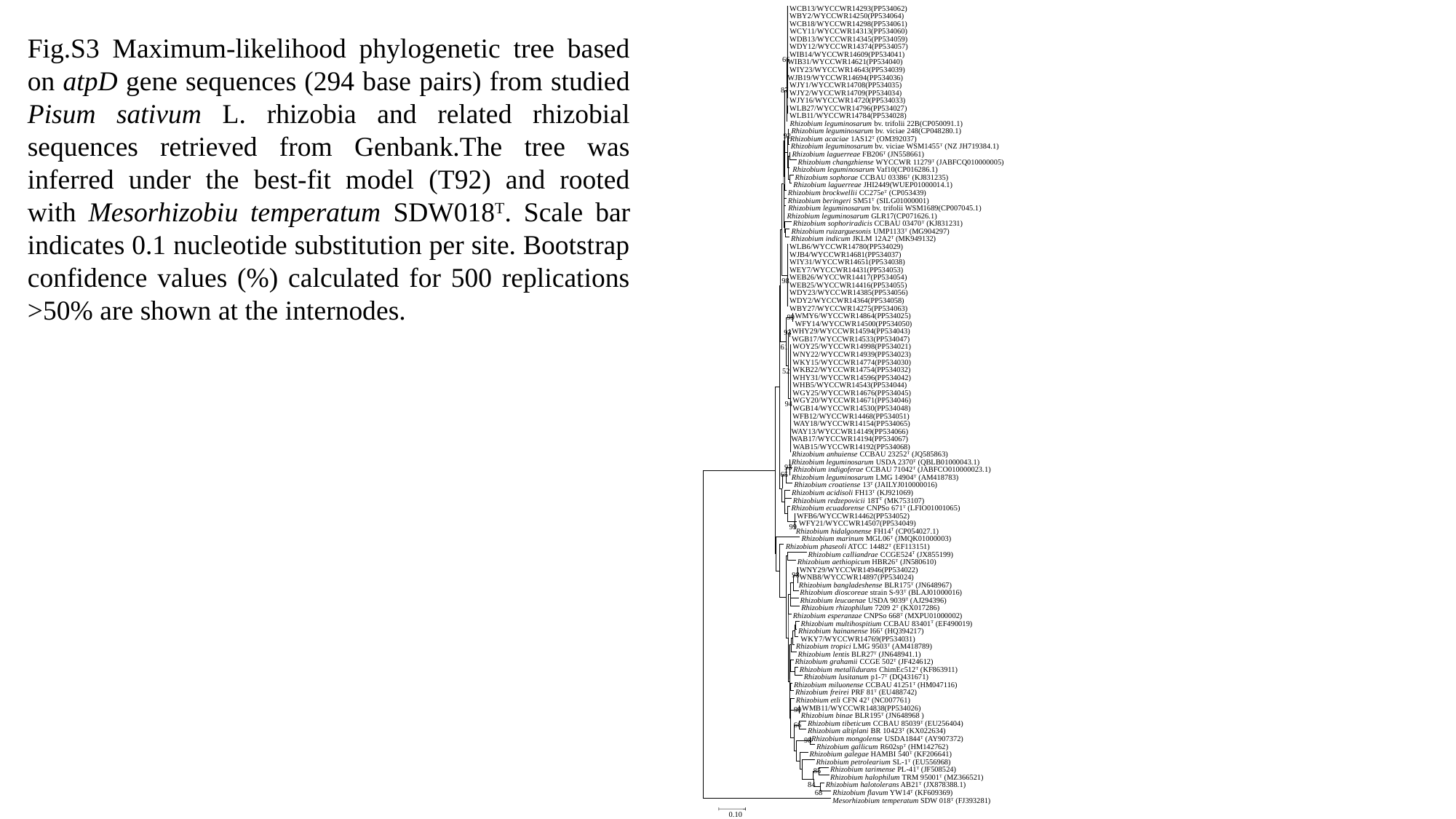

WCB13/WYCCWR14293(PP534062)
 WBY2/WYCCWR14250(PP534064)
 WCB18/WYCCWR14298(PP534061)
 WCY11/WYCCWR14313(PP534060)
 WDB13/WYCCWR14345(PP534059)
 WDY12/WYCCWR14374(PP534057)
 WIB14/WYCCWR14609(PP534041)
WIB31/WYCCWR14621(PP534040)
 WIY23/WYCCWR14643(PP534039)
WJB19/WYCCWR14694(PP534036)
 WJY1/WYCCWR14708(PP534035)
 WJY2/WYCCWR14709(PP534034)
 WJY16/WYCCWR14720(PP534033)
 WLB27/WYCCWR14796(PP534027)
 WLB11/WYCCWR14784(PP534028)
 Rhizobium leguminosarum bv. trifolii 22B(CP050091.1)
 Rhizobium leguminosarum bv. viciae 248(CP048280.1)
 Rhizobium acaciae 1AS12T (OM392037)
 Rhizobium leguminosarum bv. viciae WSM1455T (NZ JH719384.1)
 Rhizobium laguerreae FB206T (JN558661)
 Rhizobium changzhiense WYCCWR 11279T (JABFCQ010000005)
 Rhizobium leguminosarum Vaf10(CP016286.1)
 Rhizobium sophorae CCBAU 03386T (KJ831235)
 Rhizobium laguerreae JHI2449(WUEP01000014.1)
 Rhizobium brockwellii CC275eT (CP053439)
 Rhizobium beringeri SM51T (SILG01000001)
 Rhizobium leguminosarum bv. trifolii WSM1689(CP007045.1)
 Rhizobium leguminosarum GLR17(CP071626.1)
 Rhizobium sophoriradicis CCBAU 03470T (KJ831231)
 Rhizobium ruizarguesonis UMP1133T (MG904297)
 Rhizobium indicum JKLM 12A2T (MK949132)
 WLB6/WYCCWR14780(PP534029)
 WJB4/WYCCWR14681(PP534037)
 WIY31/WYCCWR14651(PP534038)
 WEY7/WYCCWR14431(PP534053)
 WEB26/WYCCWR14417(PP534054)
 WEB25/WYCCWR14416(PP534055)
 WDY23/WYCCWR14385(PP534056)
 WDY2/WYCCWR14364(PP534058)
 WBY27/WYCCWR14275(PP534063)
 WMY6/WYCCWR14864(PP534025)
 WFY14/WYCCWR14500(PP534050)
 WHY29/WYCCWR14594(PP534043)
 WGB17/WYCCWR14533(PP534047)
 WOY25/WYCCWR14998(PP534021)
 WNY22/WYCCWR14939(PP534023)
 WKY15/WYCCWR14774(PP534030)
 WKB22/WYCCWR14754(PP534032)
 WHY31/WYCCWR14596(PP534042)
 WHB5/WYCCWR14543(PP534044)
 WGY25/WYCCWR14676(PP534045)
 WGY20/WYCCWR14671(PP534046)
 WGB14/WYCCWR14530(PP534048)
 WFB12/WYCCWR14468(PP534051)
 WAY18/WYCCWR14154(PP534065)
WAY13/WYCCWR14149(PP534066)
WAB17/WYCCWR14194(PP534067)
 WAB15/WYCCWR14192(PP534068)
 Rhizobium anhuiense CCBAU 23252T (JQ585863)
 Rhizobium leguminosarum USDA 2370T (QBLB01000043.1)
 Rhizobium indigoferae CCBAU 71042T (JABFCO010000023.1)
 Rhizobium leguminosarum LMG 14904T (AM418783)
 Rhizobium croatiense 13T (JAILYJ010000016)
 Rhizobium acidisoli FH13T (KJ921069)
 Rhizobium redzepovicii 18TT (MK753107)
 Rhizobium ecuadorense CNPSo 671T (LFIO01001065)
 WFB6/WYCCWR14462(PP534052)
 WFY21/WYCCWR14507(PP534049)
 Rhizobium hidalgonense FH14T (CP054027.1)
 Rhizobium marinum MGL06T (JMQK01000003)
 Rhizobium phaseoli ATCC 14482T (EF113151)
 Rhizobium calliandrae CCGE524T (JX855199)
 Rhizobium aethiopicum HBR26T (JN580610)
 WNY29/WYCCWR14946(PP534022)
 WNB8/WYCCWR14897(PP534024)
 Rhizobium bangladeshense BLR175T (JN648967)
 Rhizobium dioscoreae strain S-93T (BLAJ01000016)
 Rhizobium leucaenae USDA 9039T (AJ294396)
 Rhizobium rhizophilum 7209 2T (KX017286)
66
87
92
98
99
93
61
52
94
93
65
99
98
 Rhizobium esperanzae CNPSo 668T (MXPU01000002)
 Rhizobium multihospitium CCBAU 83401T (EF490019)
 Rhizobium hainanense I66T (HQ394217)
 WKY7/WYCCWR14769(PP534031)
 Rhizobium tropici LMG 9503T (AM418789)
 Rhizobium lentis BLR27T (JN648941.1)
 Rhizobium grahamii CCGE 502T (JF424612)
 Rhizobium metallidurans ChimEc512T (KF863911)
 Rhizobium lusitanum p1-7T (DQ431671)
 Rhizobium miluonense CCBAU 41251T (HM047116)
 Rhizobium freirei PRF 81T (EU488742)
 Rhizobium etli CFN 42T (NC007761)
 WMB11/WYCCWR14838(PP534026)
99
 Rhizobium binae BLR195T (JN648968 )
 Rhizobium tibeticum CCBAU 85039T (EU256404)
66
 Rhizobium altiplani BR 10423T (KX022634)
 Rhizobium mongolense USDA1844T (AY907372)
98
 Rhizobium gallicum R602spT (HM142762)
 Rhizobium galegae HAMBI 540T (KF206641)
 Rhizobium petrolearium SL-1T (EU556968)
 Rhizobium tarimense PL-41T (JF508524)
85
 Rhizobium halophilum TRM 95001T (MZ366521)
84
 Rhizobium halotolerans AB21T (JX878388.1)
68
 Rhizobium flavum YW14T (KF609369)
 Mesorhizobium temperatum SDW 018T (FJ393281)
0.10
Fig.S3 Maximum-likelihood phylogenetic tree based on atpD gene sequences (294 base pairs) from studied Pisum sativum L. rhizobia and related rhizobial sequences retrieved from Genbank.The tree was inferred under the best-fit model (T92) and rooted with Mesorhizobiu temperatum SDW018T. Scale bar indicates 0.1 nucleotide substitution per site. Bootstrap confidence values (%) calculated for 500 replications >50% are shown at the internodes.

## Slide 4
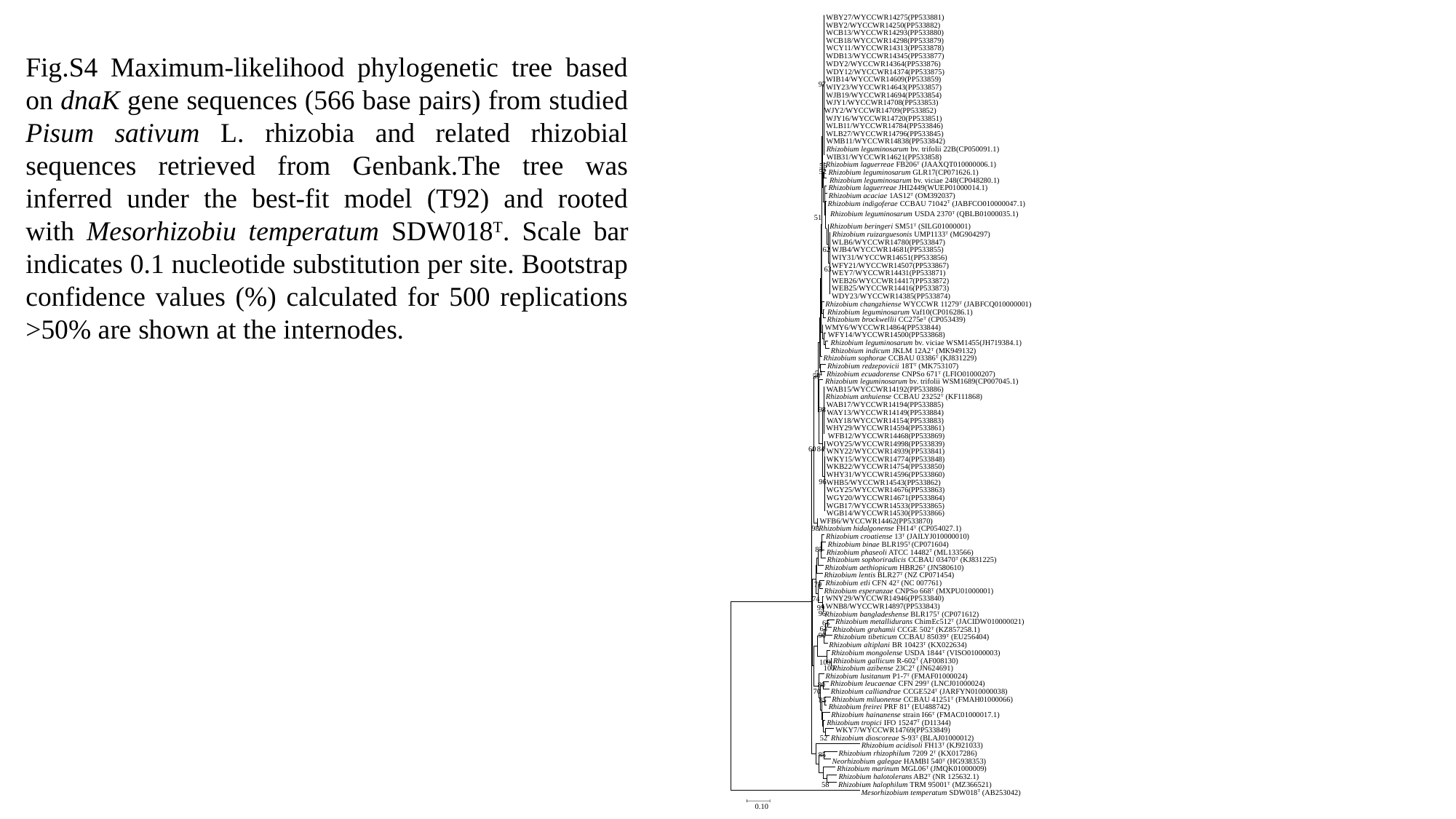

WBY27/WYCCWR14275(PP533881)
 WBY2/WYCCWR14250(PP533882)
 WCB13/WYCCWR14293(PP533880)
 WCB18/WYCCWR14298(PP533879)
 WCY11/WYCCWR14313(PP533878)
 WDB13/WYCCWR14345(PP533877)
 WDY2/WYCCWR14364(PP533876)
 WDY12/WYCCWR14374(PP533875)
 WIB14/WYCCWR14609(PP533859)
 WIY23/WYCCWR14643(PP533857)
 WJB19/WYCCWR14694(PP533854)
 WJY1/WYCCWR14708(PP533853)
WJY2/WYCCWR14709(PP533852)
 WJY16/WYCCWR14720(PP533851)
 WLB11/WYCCWR14784(PP533846)
 WLB27/WYCCWR14796(PP533845)
 WMB11/WYCCWR14838(PP533842)
 Rhizobium leguminosarum bv. trifolii 22B(CP050091.1)
 WIB31/WYCCWR14621(PP533858)
 Rhizobium laguerreae FB206T (JAAXQT010000006.1)
 Rhizobium leguminosarum GLR17(CP071626.1)
 Rhizobium leguminosarum bv. viciae 248(CP048280.1)
 Rhizobium laguerreae JHI2449(WUEP01000014.1)
 Rhizobium acaciae 1AS12T (OM392037)
 Rhizobium indigoferae CCBAU 71042T (JABFCO010000047.1)
 Rhizobium leguminosarum USDA 2370T (QBLB01000035.1)
 Rhizobium beringeri SM51T (SILG01000001)
 Rhizobium ruizarguesonis UMP1133T (MG904297)
 WLB6/WYCCWR14780(PP533847)
 WJB4/WYCCWR14681(PP533855)
 WIY31/WYCCWR14651(PP533856)
 WFY21/WYCCWR14507(PP533867)
 WEY7/WYCCWR14431(PP533871)
 WEB26/WYCCWR14417(PP533872)
 WEB25/WYCCWR14416(PP533873)
 WDY23/WYCCWR14385(PP533874)
 Rhizobium changzhiense WYCCWR 11279T (JABFCQ010000001)
 Rhizobium leguminosarum Vaf10(CP016286.1)
 Rhizobium brockwellii CC275eT (CP053439)
 WMY6/WYCCWR14864(PP533844)
 WFY14/WYCCWR14500(PP533868)
 Rhizobium leguminosarum bv. viciae WSM1455(JH719384.1)
 Rhizobium indicum JKLM 12A2T (MK949132)
 Rhizobium sophorae CCBAU 03386T (KJ831229)
 Rhizobium redzepovicii 18TT (MK753107)
 Rhizobium ecuadorense CNPSo 671T (LFIO01000207)
 Rhizobium leguminosarum bv. trifolii WSM1689(CP007045.1)
 WAB15/WYCCWR14192(PP533886)
 Rhizobium anhuiense CCBAU 23252T (KF111868)
 WAB17/WYCCWR14194(PP533885)
 WAY13/WYCCWR14149(PP533884)
 WAY18/WYCCWR14154(PP533883)
 WHY29/WYCCWR14594(PP533861)
 WFB12/WYCCWR14468(PP533869)
 WOY25/WYCCWR14998(PP533839)
 WNY22/WYCCWR14939(PP533841)
 WKY15/WYCCWR14774(PP533848)
 WKB22/WYCCWR14754(PP533850)
 WHY31/WYCCWR14596(PP533860)
 WHB5/WYCCWR14543(PP533862)
 WGY25/WYCCWR14676(PP533863)
 WGY20/WYCCWR14671(PP533864)
 WGB17/WYCCWR14533(PP533865)
 WGB14/WYCCWR14530(PP533866)
 WFB6/WYCCWR14462(PP533870)
 Rhizobium hidalgonense FH14T (CP054027.1)
 Rhizobium croatiense 13T (JAILYJ010000010)
 Rhizobium binae BLR195T (CP071604)
 Rhizobium phaseoli ATCC 14482T (ML133566)
 Rhizobium sophoriradicis CCBAU 03470T (KJ831225)
 Rhizobium aethiopicum HBR26T (JN580610)
 Rhizobium lentis BLR27T (NZ CP071454)
 Rhizobium etli CFN 42T (NC 007761)
 Rhizobium esperanzae CNPSo 668T (MXPU01000001)
 WNY29/WYCCWR14946(PP533840)
 WNB8/WYCCWR14897(PP533843)
 Rhizobium bangladeshense BLR175T (CP071612)
97
55
52
51
62
63
54
59
98
84
60
96
98
85
79
74
99
96
 Rhizobium metallidurans ChimEc512T (JACIDW010000021)
65
63
 Rhizobium grahamii CCGE 502T (KZ857258.1)
90
 Rhizobium tibeticum CCBAU 85039T (EU256404)
 Rhizobium altiplani BR 10423T (KX022634)
 Rhizobium mongolense USDA 1844T (VISO01000003)
 Rhizobium gallicum R-602T (AF008130)
100
100
 Rhizobium azibense 23C2T (JN624691)
 Rhizobium lusitanum P1-7T (FMAF01000024)
 Rhizobium leucaenae CFN 299T (LNCJ01000024)
80
 Rhizobium calliandrae CCGE524T (JARFYN010000038)
70
 Rhizobium miluonense CCBAU 41251T (FMAH01000066)
85
 Rhizobium freirei PRF 81T (EU488742)
 Rhizobium hainanense strain I66T (FMAC01000017.1)
 Rhizobium tropici IFO 15247T (D11344)
 WKY7/WYCCWR14769(PP533849)
52
 Rhizobium dioscoreae S-93T (BLAJ01000012)
 Rhizobium acidisoli FH13T (KJ921033)
 Rhizobium rhizophilum 7209 2T (KX017286)
85
 Neorhizobium galegae HAMBI 540T (HG938353)
 Rhizobium marinum MGL06T (JMQK01000009)
 Rhizobium halotolerans AB2T (NR 125632.1)
58
 Rhizobium halophilum TRM 95001T (MZ366521)
 Mesorhizobium temperatum SDW018T (AB253042)
0.10
Fig.S4 Maximum-likelihood phylogenetic tree based on dnaK gene sequences (566 base pairs) from studied Pisum sativum L. rhizobia and related rhizobial sequences retrieved from Genbank.The tree was inferred under the best-fit model (T92) and rooted with Mesorhizobiu temperatum SDW018T. Scale bar indicates 0.1 nucleotide substitution per site. Bootstrap confidence values (%) calculated for 500 replications >50% are shown at the internodes.

## Slide 5
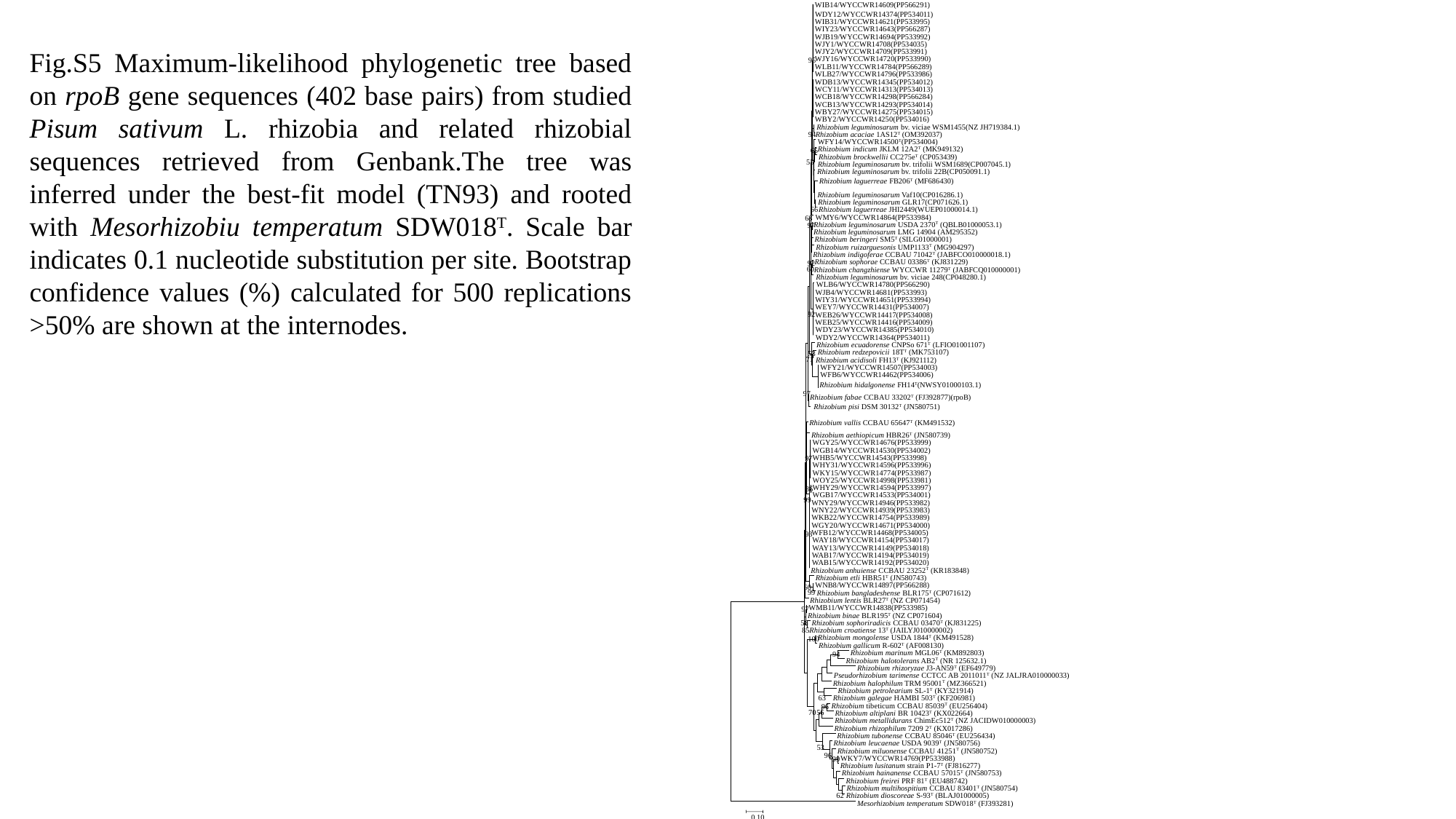

WIB14/WYCCWR14609(PP566291)
 WDY12/WYCCWR14374(PP534011)
 WIB31/WYCCWR14621(PP533995)
 WIY23/WYCCWR14643(PP566287)
 WJB19/WYCCWR14694(PP533992)
 WJY1/WYCCWR14708(PP534035)
 WJY2/WYCCWR14709(PP533991)
 WJY16/WYCCWR14720(PP533990)
 WLB11/WYCCWR14784(PP566289)
 WLB27/WYCCWR14796(PP533986)
 WDB13/WYCCWR14345(PP534012)
 WCY11/WYCCWR14313(PP534013)
 WCB18/WYCCWR14298(PP566284)
 WCB13/WYCCWR14293(PP534014)
 WBY27/WYCCWR14275(PP534015)
 WBY2/WYCCWR14250(PP534016)
 Rhizobium leguminosarum bv. viciae WSM1455(NZ JH719384.1)
 Rhizobium acaciae 1AS12T (OM392037)
 WFY14/WYCCWR14500T(PP534004)
 Rhizobium indicum JKLM 12A2T (MK949132)
 Rhizobium brockwellii CC275eT (CP053439)
 Rhizobium leguminosarum bv. trifolii WSM1689(CP007045.1)
 Rhizobium leguminosarum bv. trifolii 22B(CP050091.1)
 Rhizobium laguerreae FB206T (MF686430)
 Rhizobium leguminosarum Vaf10(CP016286.1)
 Rhizobium leguminosarum GLR17(CP071626.1)
 Rhizobium laguerreae JHI2449(WUEP01000014.1)
 WMY6/WYCCWR14864(PP533984)
 Rhizobium leguminosarum USDA 2370T (QBLB01000053.1)
 Rhizobium leguminosarum LMG 14904 (AM295352)
 Rhizobium beringeri SM5T (SILG01000001)
 Rhizobium ruizarguesonis UMP1133T (MG904297)
 Rhizobium indigoferae CCBAU 71042T (JABFCO010000018.1)
 Rhizobium sophorae CCBAU 03386T (KJ831229)
 Rhizobium changzhiense WYCCWR 11279T (JABFCQ010000001)
 Rhizobium leguminosarum bv. viciae 248(CP048280.1)
 WLB6/WYCCWR14780(PP566290)
 WJB4/WYCCWR14681(PP533993)
 WIY31/WYCCWR14651(PP533994)
 WEY7/WYCCWR14431(PP534007)
 WEB26/WYCCWR14417(PP534008)
 WEB25/WYCCWR14416(PP534009)
 WDY23/WYCCWR14385(PP534010)
 WDY2/WYCCWR14364(PP534011)
 Rhizobium ecuadorense CNPSo 671T (LFIO01001107)
 Rhizobium redzepovicii 18TT (MK753107)
 Rhizobium acidisoli FH13T (KJ921112)
 WFY21/WYCCWR14507(PP534003)
 WFB6/WYCCWR14462(PP534006)
 Rhizobium hidalgonense FH14T(NWSY01000103.1)
 Rhizobium fabae CCBAU 33202T (FJ392877)(rpoB)
 Rhizobium pisi DSM 30132T (JN580751)
 Rhizobium vallis CCBAU 65647T (KM491532)
 Rhizobium aethiopicum HBR26T (JN580739)
 WGY25/WYCCWR14676(PP533999)
 WGB14/WYCCWR14530(PP534002)
 WHB5/WYCCWR14543(PP533998)
 WHY31/WYCCWR14596(PP533996)
 WKY15/WYCCWR14774(PP533987)
 WOY25/WYCCWR14998(PP533981)
 WHY29/WYCCWR14594(PP533997)
 WGB17/WYCCWR14533(PP534001)
 WNY29/WYCCWR14946(PP533982)
 WNY22/WYCCWR14939(PP533983)
 WKB22/WYCCWR14754(PP533989)
 WGY20/WYCCWR14671(PP534000)
 WFB12/WYCCWR14468(PP534005)
 WAY18/WYCCWR14154(PP534017)
 WAY13/WYCCWR14149(PP534018)
 WAB17/WYCCWR14194(PP534019)
 WAB15/WYCCWR14192(PP534020)
 Rhizobium anhuiense CCBAU 23252T (KR183848)
 Rhizobium etli HBR51T (JN580743)
 WNB8/WYCCWR14897(PP566288)
95
94
65
55
66
68
97
92
60
92
53
71
97
97
86
99
93
60
99
 Rhizobium bangladeshense BLR175T (CP071612)
 Rhizobium lentis BLR27T (NZ CP071454)
 WMB11/WYCCWR14838(PP533985)
97
 Rhizobium binae BLR195T (NZ CP071604)
58
 Rhizobium sophoriradicis CCBAU 03470T (KJ831225)
85
 Rhizobium croatiense 13T (JAILYJ010000002)
 Rhizobium mongolense USDA 1844T (KM491528)
100
 Rhizobium gallicum R-602T (AF008130)
 Rhizobium marinum MGL06T (KM892803)
94
 Rhizobium halotolerans AB2T (NR 125632.1)
 Rhizobium rhizoryzae J3-AN59T (EF649779)
 Pseudorhizobium tarimense CCTCC AB 2011011T (NZ JALJRA010000033)
 Rhizobium halophilum TRM 95001T (MZ366521)
 Rhizobium petrolearium SL-1T (KY321914)
63
 Rhizobium galegae HAMBI 503T (KF206981)
 Rhizobium tibeticum CCBAU 85039T (EU256404)
96
56
70
 Rhizobium altiplani BR 10423T (KX022664)
 Rhizobium metallidurans ChimEc512T (NZ JACIDW010000003)
 Rhizobium rhizophilum 7209 2T (KX017286)
 Rhizobium tubonense CCBAU 85046T (EU256434)
 Rhizobium leucaenae USDA 9039T (JN580756)
53
 Rhizobium miluonense CCBAU 41251T (JN580752)
96
 WKY7/WYCCWR14769(PP533988)
99
 Rhizobium lusitanum strain P1-7T (FJ816277)
 Rhizobium hainanense CCBAU 57015T (JN580753)
 Rhizobium freirei PRF 81T (EU488742)
 Rhizobium multihospitium CCBAU 83401T (JN580754)
62
 Rhizobium dioscoreae S-93T (BLAJ01000005)
 Mesorhizobium temperatum SDW018T (FJ393281)
0.10
Fig.S5 Maximum-likelihood phylogenetic tree based on rpoB gene sequences (402 base pairs) from studied Pisum sativum L. rhizobia and related rhizobial sequences retrieved from Genbank.The tree was inferred under the best-fit model (TN93) and rooted with Mesorhizobiu temperatum SDW018T. Scale bar indicates 0.1 nucleotide substitution per site. Bootstrap confidence values (%) calculated for 500 replications >50% are shown at the internodes.

## Slide 6
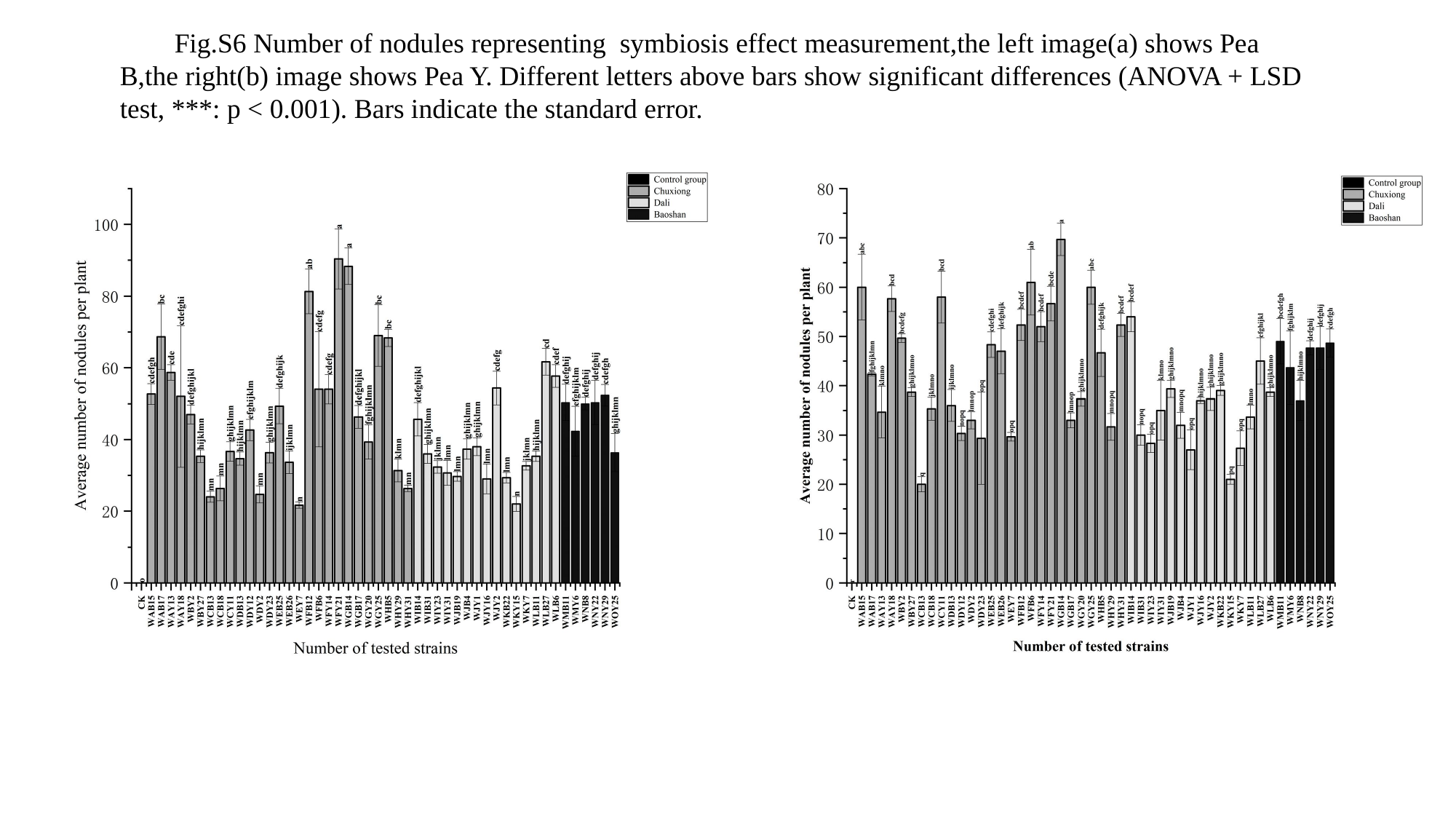

Fig.S6 Number of nodules representing symbiosis effect measurement,the left image(a) shows Pea B,the right(b) image shows Pea Y. Different letters above bars show significant differences (ANOVA + LSD test, ***: p < 0.001). Bars indicate the standard error.

## Slide 7
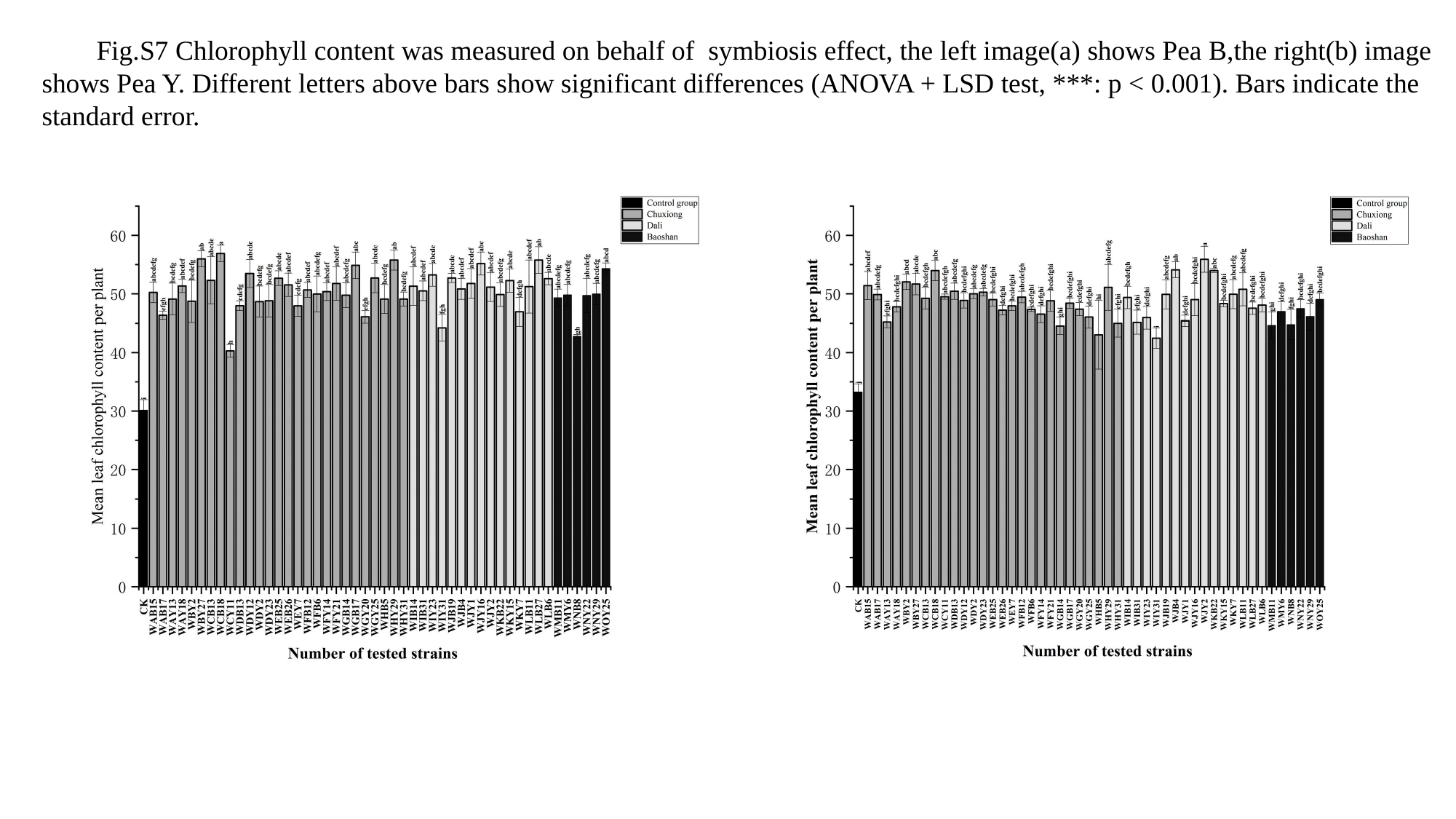

Fig.S7 Chlorophyll content was measured on behalf of symbiosis effect, the left image(a) shows Pea B,the right(b) image shows Pea Y. Different letters above bars show significant differences (ANOVA + LSD test, ***: p < 0.001). Bars indicate the standard error.

## Slide 8
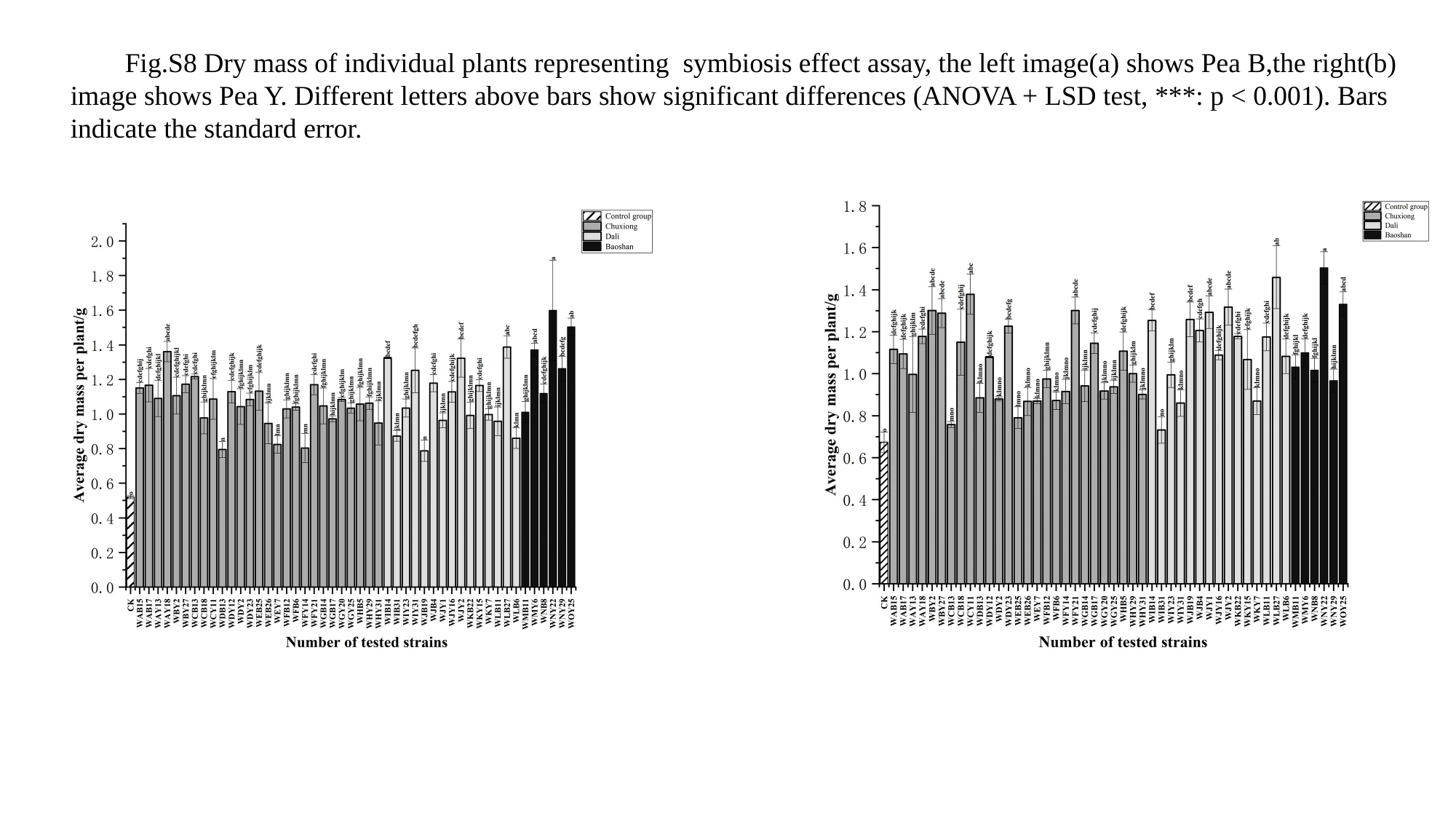

Fig.S8 Dry mass of individual plants representing symbiosis effect assay, the left image(a) shows Pea B,the right(b) image shows Pea Y. Different letters above bars show significant differences (ANOVA + LSD test, ***: p < 0.001). Bars indicate the standard error.

## Slide 9
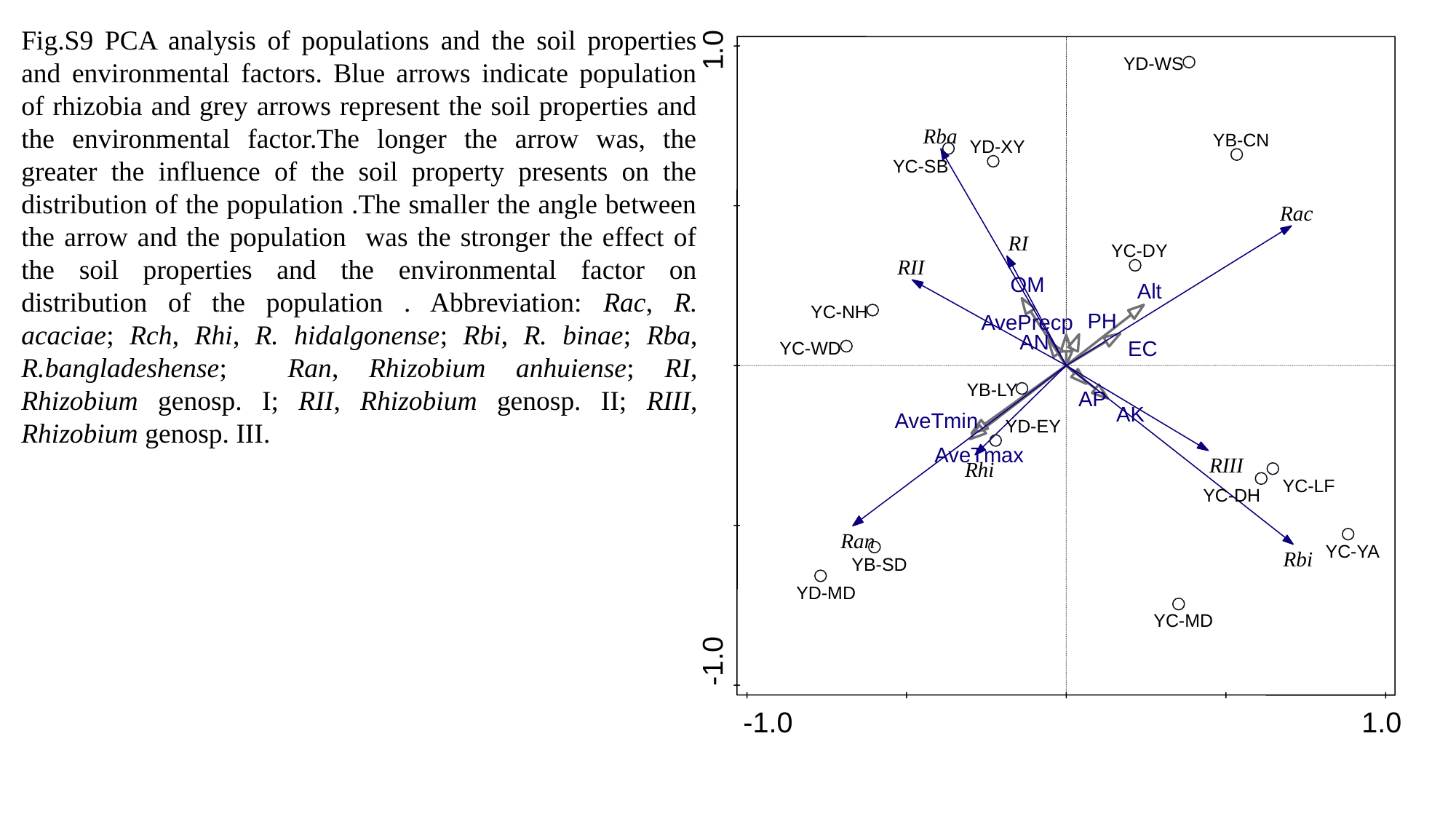

Fig.S9 PCA analysis of populations and the soil properties and environmental factors. Blue arrows indicate population of rhizobia and grey arrows represent the soil properties and the environmental factor.The longer the arrow was, the greater the influence of the soil property presents on the distribution of the population .The smaller the angle between the arrow and the population was the stronger the effect of the soil properties and the environmental factor on distribution of the population . Abbreviation: Rac, R. acaciae; Rch, Rhi, R. hidalgonense; Rbi, R. binae; Rba, R.bangladeshense; Ran, Rhizobium anhuiense; RI, Rhizobium genosp. I; RII, Rhizobium genosp. II; RIII, Rhizobium genosp. III.
1.0
YD-WS
Rba
YB-CN
YD-XY
YC-SB
Rac
RI
YC-DY
RII
OM
Alt
YC-NH
PH
AvePrecp
AN
EC
YC-WD
YB-LY
AP
AK
AveTmin
YD-EY
AveTmax
RIII
Rhi
YC-LF
YC-DH
Ran
YC-YA
Rbi
YB-SD
YD-MD
YC-MD
-1.0
-1.0
1.0

## Slide 10
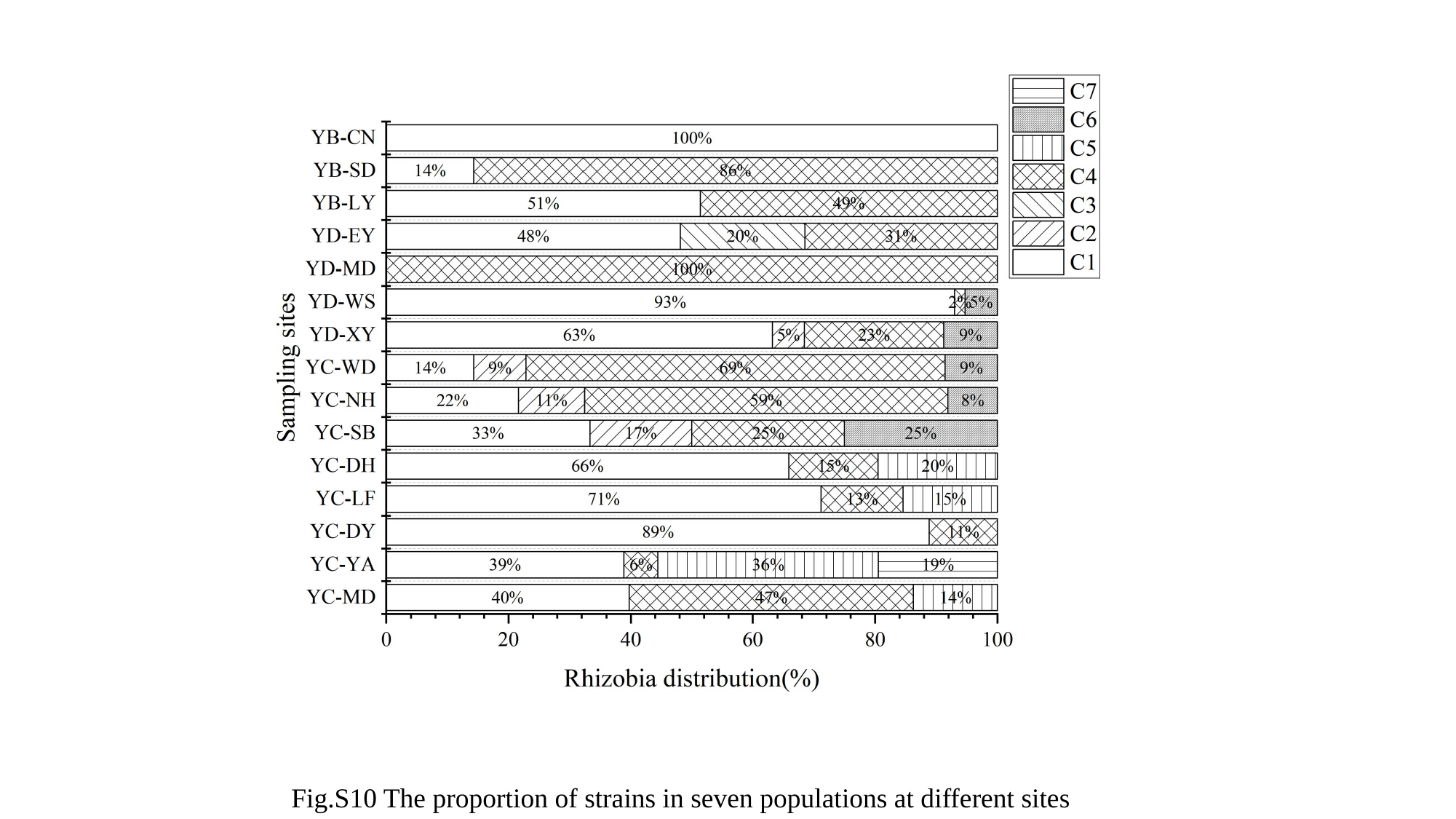

Fig.S10 The proportion of strains in seven populations at different sites

## Slide 11
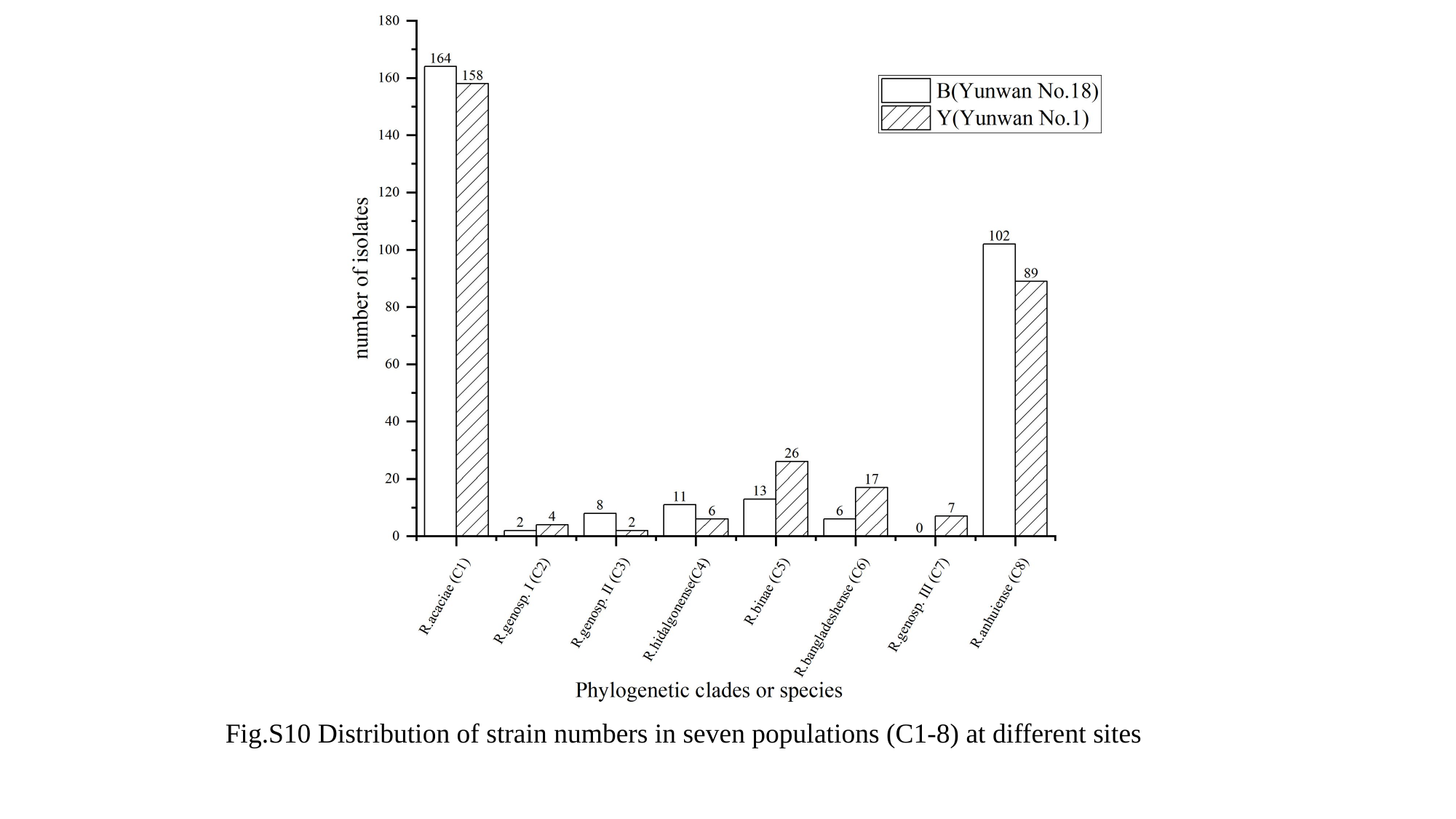

Fig.S10 Distribution of strain numbers in seven populations (C1-8) at different sites
