# Supplementary material for: Rhizobiumacaciae and R. anhuiense are the dominant rhizobial symbionts of Pisum sativum L. from Yunnan-Guizhou Plateau
Source: Front Microbiol. 2024 Sep 26;15:1437586. doi: 10.3389/fmicb.2024.1437586 (PMC11464311; doi:10.3389/fmicb.2024.1437586)
Supplement: Supplementary file 2 [file Table_1.DOCX]

**TABLE S1** Climatological data from different sampling sites

| Sampling site | Latitude and  longitude | altitude（m） | Ave. Tmin℃ | Ave. Tmax℃ | Ave.  Precipitation  （mm） |
| --- | --- | --- | --- | --- | --- |
| YC-MD | N101.9167  E25.4833 | 2022 | 10.0 | 21.5 | 76.8 |
| YC-YA | N101.3833  E26.0167 | 2569 | 6.7 | 18.3 | 85.2 |
| YC-DY | N101.4667  E26.0333 | 2234 | 8.6 | 20.4 | 81.4 |
| YC-LF | N102.5  E25.3 | 1816 | 10.8 | 21.9 | 76.2 |
| YC-DH | N101.4333  E24.9333 | 1925 | 11.0 | 22.4 | 78.3 |
| YC-SB | N101.85  E24.75 | 1583 | 13.2 | 24.5 | 72.5 |
| YC-NH | N101.5  E25.1167 | 1861 | 11.3 | 22.8 | 75.5 |
| YC-WD | N101.0833  E26.0833 | 2967 | 4.5 | 15.9 | 87.6 |
| YD-XY | N100.607  E25.4649 | 2013 | 10.1 | 21.4 | 81.6 |
| YD-WS | N100.2097  E25.4025 | 1770 | 11.2 | 22.4 | 82.1 |
| YD-MD | N100.8714  E25.4786 | 1992 | 10.3 | 21.7 | 79.3 |
| YD-EY | N99.9668  E26.2324 | 2106 | 9.3 | 20.6 | 85.9 |
| YB-LY | N98.8352  E25.0591 | 1047 | 13.6 | 25.1 | 112.7 |
| YB-SD | N99.2394  E24.571 | 1864 | 9.6 | 21.1 | 96.8 |
| YB-CN | N99.447  E24.8081 | 1033 | 14.1 | 25.8 | 95.4 |

Ave. Tmin is average annual minimum air temperature; Ave. Tmax is Average annual maximum air temperature; Ave. Precipitation is average annual climate and humidity.

**TABLE S2** Soil properties from the different sites studied

| Sampling  Site^#^ | Properties of soil* | | | | | |
| --- | --- | --- | --- | --- | --- | --- |
|  | pH | Ec (μs/cm) | OM (g/kg) | EN (mg/kg) | AP (mg/kg) | AK (mg/kg) |
| YC-MD | 5.47±0.02 l | 117±2.65 e | 28.93±0.75 g | 126±7.80 efg | 77.03±1.50 d | 358.33±5.03 a |
| YC-YA | 7.74±0.03 a | 245±38.43 c | 25.17±0.40 i | 146±21.66 bcdef | 23.2±2.34 kl | 132.67±1.53 g |
| YC-DY | 7.42±0.01 cd | 819.33±33.50 a | 30.23±0.32 f | 151.93±8.01 bcde | 26.37±0.60 j | 97.57±0.95 j |
| YC-LF | 7.22±0.04 ef | 142.67±8.50 de | 32.57±0.55 e | 167.33±14.55 bc | 37.83±0.55 h | 132.33±1.53 g |
| YC-DH | 5.77±0.04 k | 74.37±12.36 f | 24.8±0.35 i | 103.17±3.34 g | 22.93±0.40 kl | 152.33±0.58 e |
| YC-SB | 7.24±0.03 ef | 123.33±10.02 e | 36.2±0.17 c | 172.47±15.54 b | 35.1±0.72 i | 200±1.73 d |
| YC-NH | 6.37±0.01 i | 80.73±10.71 f | 30.73±0.45 f | 136.03±4.97 cdef | 12.57±0.23 m | 108.67±2.08 i |
| YC-WD | 5.43±0.01 l | 76.67±2.53 f | 33.97±0.31 d | 141.7±6.64 bcdef | 21.73±0.42 kl | 117.67±0.58 h |
| YD-XY | 7.02±0.02 g | 170.33±30.86 d | 39.2±0.36 b | 150.7±14.85 bcde | 20.03±0.06 l | 212±1.73 c |
| YD-WS | 7.35±0.02 d | 126±1.00 e | 32.53±0.38 e | 159.63±6.21 bcd | 46.13±2.20 g | 106±1.00 i |
| YD-MD | 6.93±0.01 h | 69.13±6.56 f | 29.1±0.26 g | 133.47±24.50 defg | 93.8±0.56 b | 144.67±2.08 f |
| YD-EY | 7.38±0.10 cd | 364±25.24 b | 62.6±0.87 a | 388.87±45.41 a | 60.47±0.74 e | 143.67±3.51 f |
| YB-LY | 7.16±0.03 f | 138.33±9.50 de | 19.43±0.31 j | 124.5±5.86 efg | 80.17±1.76 c | 345±2.00 b |
| YB-SD | 7.52±0.02 b | 162.67±15.70 d | 17.2±0.52 k | 118.1±5.86 fg | 49.33±1.50 f | 88.7±1.00 k |
| YB-CN | 6.07±0.01 j | 120±11.14 e | 28±0.70 h | 166.87±5.90 bc | 138.93±2.25 a | 214.67±4.04 c |

*. OM is organic matter; EN is effective nitrogen; AP is available phosphate; AK is available potassium; Ec is conductivity.

#. YC-MD refers to Mouding County, Chuxiong city; YC-YA refers to Yaoan county, Chuxiong city; YC-DY refers to Dayao county, Chuxiong city; YC-LF refers to Lufeng city, Chuxiong city; YC-DH refers to Donghua town, Chuxiong city; YC-WD refers to Wuding County, Chuxiong city; YC-SP refers to Shuangpai county, Chuxiong city; YC-NH refers to Nanhua county, Chuxiong city; YD-XY refers to Xiangyun county, Dali city; YD-WS refers to Weishan county, Dali city; YD-MD refers to Midu county, Dali city; YD-EY refers to Eryuan county, Dali city; YB-LY refers to Longyang district, Baoshan city; YB-CN refers to Changning county, Baoshan city; YB-SD refers to Shidian county, Baoshan city;

Data in the table are the mean value and its standard deviation (three replicates). Different letters in a column indicated that the difference between different soil properties reached a 0.05 significant level (P≤0.05).

**TABLE S3** Symbiotic data of the pea B and pea Y by inoculation of each *Rhizobium* isolate.

| Isolates | Average of total dry weight/g | | Average number of nodules | | Average chlorophyll content | |
| --- | --- | --- | --- | --- | --- | --- |
|  | Pea B | Pea Y | Pea B | Pea Y | Pea B | Pea Y |
| Negative control | 0.520 | 0.674 | 0 | 0 | 30.13 | 33.23 |
| WAB15 | 1.151 | 1.116 | 53 | 60 | 50.23 | 51.40 |
| WAB17 | 1.167 | 1.094 | 69 | 42 | 46.33 | 49.87 |
| WAY13 | 1.090 | 0.996 | 59 | 35 | 49.10 | 45.23 |
| WAY18 | 1.361 | 1.177 | 52 | 58 | 51.33 | 47.77 |
| WBY2 | 1.106 | 1.301 | 47 | 50 | 48.73 | 52.03 |
| WBY27 | 1.173 | 1.288 | 35 | 39 | 55.97 | 51.67 |
| WCB13 | 1.217 | 0.758 | 24 | 20 | 52.30 | 49.23 |
| WCB18 | 0.977 | 1.150 | 26 | 35 | 56.90 | 53.97 |
| WCY11 | 1.087 | 1.378 | 37 | 58 | 40.30 | 49.50 |
| WDB13 | 0.795 | 0.884 | 35 | 36 | 47.97 | 50.43 |
| WDY12 | 1.130 | 1.079 | 43 | 30 | 53.47 | 48.87 |
| WDY2 | 1.043 | 0.879 | 25 | 33 | 48.67 | 50.00 |
| WDY23 | 1.084 | 1.226 | 36 | 29 | 48.80 | 50.27 |
| WEB25 | 1.132 | 0.791 | 49 | 48 | 52.67 | 49.00 |
| WEB26 | 0.945 | 0.867 | 34 | 47 | 51.53 | 47.20 |
| WEY7 | 0.824 | 0.869 | 22 | 30 | 47.93 | 47.93 |
| WFB12 | 1.030 | 0.974 | 81 | 52 | 50.67 | 49.43 |
| WFB6 | 1.041 | 0.871 | 54 | 61 | 49.97 | 47.33 |
| WFY14 | 0.803 | 0.914 | 54 | 52 | 50.37 | 46.53 |
| WFY21 | 1.170 | 1.301 | 90 | 57 | 51.77 | 48.80 |
| WGB14 | 1.047 | 0.941 | 88 | 70 | 49.77 | 44.53 |
| WGB17 | 0.972 | 1.145 | 46 | 33 | 54.87 | 48.40 |
| WGY20 | 1.084 | 0.917 | 39 | 37 | 46.07 | 47.37 |
| WGY25 | 1.034 | 0.937 | 69 | 60 | 52.70 | 46.03 |
| WHB5 | 1.058 | 1.107 | 68 | 47 | 49.10 | 43.03 |
| WHY29 | 1.063 | 1.000 | 31 | 32 | 55.77 | 51.10 |
| WHY31 | 0.948 | 0.901 | 26 | 52 | 49.10 | 45.00 |
| WIB14 | 1.325 | 1.254 | 46 | 54 | 51.30 | 49.37 |
| WIB31 | 0.873 | 0.733 | 36 | 30 | 50.50 | 45.17 |
| WIY23 | 1.034 | 0.994 | 32 | 28 | 53.23 | 45.93 |
| WIY31 | 1.253 | 0.859 | 31 | 35 | 44.23 | 42.47 |
| WJB19 | 0.787 | 1.258 | 30 | 39 | 52.70 | 49.90 |
| WJB4 | 1.179 | 1.206 | 37 | 32 | 50.83 | 54.10 |
| WJY1 | 0.963 | 1.293 | 38 | 27 | 51.77 | 45.40 |
| WJY16 | 1.128 | 1.088 | 29 | 37 | 55.17 | 49.00 |
| WJY2 | 1.323 | 1.317 | 54 | 37 | 51.13 | 55.90 |
| WKB22 | 0.992 | 1.177 | 29 | 39 | 49.87 | 53.97 |
| WKY15 | 1.165 | 1.067 | 22 | 21 | 52.27 | 48.33 |
| WKY7 | 0.997 | 0.869 | 33 | 27 | 46.93 | 49.93 |
| WLB11 | 0.958 | 1.175 | 35 | 34 | 51.23 | 50.77 |
| WLB27 | 1.388 | 1.459 | 62 | 45 | 55.77 | 47.53 |
| WLB6 | 0.860 | 1.082 | 58 | 39 | 52.60 | 48.07 |
| WMB11 | 1.011 | 1.031 | 50 | 49 | 49.30 | 44.63 |
| WMY6 | 1.372 | 1.099 | 42 | 44 | 49.80 | 46.97 |
| WNB8 | 1.119 | 1.016 | 50 | 37 | 42.77 | 44.77 |
| WNY22 | 1.598 | 1.503 | 50 | 48 | 49.70 | 47.47 |
| WNY29 | 1.263 | 0.966 | 52 | 48 | 49.97 | 46.10 |
| WOY25 | 1.503 | 1.331 | 36 | 49 | 54.30 | 49.03 |

**TABLE S4** Genetic groupings of *Rhizobium* isolates associated with *Pisum sativum* L. and their geographical distribution in the different sampling sites

| Representative isolate | Distribution (peaB/peaY) of strain numbers in the different sampling site | | | | | | | | | | | | | | |  |
| --- | --- | --- | --- | --- | --- | --- | --- | --- | --- | --- | --- | --- | --- | --- | --- | --- |
|  | YC-MD^a^ | YC-YA | YC-DY | YC-LF | YC-DH | YC-SB | YC-NH | YC-WD | YD-XY | YD-WS | YD-MD | YD-EY | YB-LY | YB-SD | YB-CN | Total |
| *Rhizobium acaciae* (Clade1,C1) (52.3%, 322 isolates, 21 IGS types, 14 sites) | | | | | | | | | | | | | | | | |
| WDB13 | 0(0/0) | 0(0/0) | 0(0/0) | 0(0/0) | 0(0/0) | 0(0/0) | 0(0/0) | 0(0/0) | 8(2/6) | 2(2/0) | 0(0/0) | 0(0/0) | 0(0/0) | 0(0/0) | 0(0/0) | 10(4/6) |
| WCY11 | 0(0/0) | 0(0/0) | 0(0/0) | 0(0/0) | 0(0/0) | 0(0/0) | 0(0/0) | 0(0/0) | 0(0/0) | 0(0/0) | 0(0/0) | 0(0/0) | 9(6/3) | 0(0/0) | 0(0/0) | 9(6/3) |
| WDY2 | 0(0/0) | 0(0/0) | 0(0/0) | 0(0/0) | 0(0/0) | 0(0/0) | 0(0/0) | 0(0/0) | 0(0/0) | 0(0/0) | 0(0/0) | 13(3/10) | 0(0/0) | 0(0/0) | 0(0/0) | 13(3/10) |
| WIB14 | 0(0/0) | 10(4/6) | 8(8/0) | 3(3/0) | 0(0/0) | 0(0/0) | 0(0/0) | 0(0/0) | 0(0/0) | 0(0/0) | 0(0/0) | 0(0/0) | 0(0/0) | 0(0/0) | 0(0/0) | 21(15/6) |
| WIY23 | 0(0/0) | 0(0/0) | 1(1/0) | 0(0/0) | 0(0/0) | 0(0/0) | 0(0/0) | 0(0/0) | 0(0/0) | 0(0/0) | 0(0/0) | 0(0/0) | 0(0/0) | 0(0/0) | 0(0/0) | 1(1/0) |
| WJB19 | 10(10/0) | 0(0/0) | 0(0/0) | 0(0/0) | 0(0/0) | 0(0/0) | 0(0/0) | 0(0/0) | 0(0/0) | 14(8/6) | 0(0/0) | 0(0/0) | 8(8/0) | 0(0/0) | 18(16/2) | 50(42/8) |
| WJY1 | 0(0/0) | 0(0/0) | 0(0/0) | 0(0/0) | 0(0/0) | 0(0/0) | 0(0/0) | 0(0/0) | 0(0/0) | 0(0/0) | 0(0/0) | 13(2/11) | 0(0/0) | 0(0/0) | 0(0/0) | 13(2/11) |
| WJY2 | 10(5/5) | 0(0/0) | 0(0/0) | 0(0/0) | 0(0/0) | 0(0/0) | 0(0/0) | 0(0/0) | 0(0/0) | 14(8/6) | 0(0/0) | 0(0/0) | 8(8/0) | 0(0/0) | 18(16/2) | 50(42/8) |
| WJY16 | 0(0/0) | 0(0/0) | 0(0/0) | 0(0/0) | 0(0/0) | 9(4/5) | 0(0/0) | 0(0/0) | 0(0/0) | 0(0/0) | 0(0/0) | 0(0/0) | 0(0/0) | 0(0/0) | 0(0/0) | 9(4/5) |
| WLB11 | 0(0/0) | 0(0/0) | 0(0/0) | 15(4/11) | 0(0/0) | 0(0/0) | 0(0/0) | 0(0/0) | 0(0/0) | 0(0/0) | 0(0/0) | 0(0/0) | 0(0/0) | 0(0/0) | 0(0/0) | 15(4/11) |
| WLB27 | 0(0/0) | 0(0/0) | 0(0/0) | 0(0/0) | 0(0/0) | 0(0/0) | 3(2/1) | 5(3/2) | 0(0/0) | 4(2/2) | 0(0/0) | 0(0/0) | 0(0/0) | 0(0/0) | 0(0/0) | 12(5/7) |
| WCB13 | 0(0/0) | 0(0/0) | 0(0/0) | 11(3/8) | 0(0/0) | 0(0/0) | 0(0/0) | 0(0/0) | 0(0/0) | 0(0/0) | 0(0/0) | 0(0/0) | 0(0/0) | 0(0/0) | 0(0/0) | 11(3/8) |
| WIB31 | 0(0/0) | 0(0/0) | 0(0/0) | 0(0/0) | 0(0/0) | 0(0/0) | 0(0/0) | 0(0/0) | 16(10/6) | 27(12/15) | 0(0/0) | 0(0/0) | 0(0/0) | 0(0/0) | 0(0/0) | 43(22/21) |
| WCB18 | 0(0/0) | 0(0/0) | 0(0/0) | 0(0/0) | 0(0/0) | 3(3/0) | 5(5/0) | 0(0/0) | 0(0/0) | 0(0/0) | 0(0/0) | 0(0/0) | 0(0/0) | 0(0/0) | 0(0/0) | 8(8/0) |
| WBY2 | 9(5/4) | 0(0/0) | 0(0/0) | 0(0/0) | 0(0/0) | 0(0/0) | 0(0/0) | 0(0/0) | 0(0/0) | 0(0/0) | 0(0/0) | 0(0/0) | 0(0/0) | 0(0/0) | 10(0/10) | 19(9/10) |
| WBY27 | 0(0/0) | 0(0/0) | 0(0/0) | 0(0/0) | 0(0/0) | 0(0/0) | 0(0/0) | 0(0/0) | 0(0/0) | 0(0/0) | 0(0/0) | 0(0/0) | 1(1/0) | 3(3/0) | 0(0/0) | 4(4/0) |
| WDY12 | 0(0/0) | 10(4/6) | 8(8/0) | 3(5/0) | 0(0/0) | 0(0/0) | 0(0/0) | 0(0/0) | 0(0/0) | 0(0/0) | 0(0/0) | 0(0/0) | 0(0/0) | 0(0/0) | 0(0/0) | 21(15/6) |
| WJB4 | 0(0/0) | 0(0/0) | 0(0/0) | 0(0/0) | 2(2/0) | 0(0/0) | 0(0/0) | 0(0/0) | 0(0/0) | 0(0/0) | 0(0/0) | 0(0/0) | 0(0/0) | 0(0/0) | 0(0/0) | 2(2/0) |
| WLB6 | 0(0/0) | 0(0/0) | 0(0/0) | 0(0/0) | 12(5/7) | 0(0/0) | 0(0/0) | 0(0/0) | 0(0/0) | 0(0/0) | 0(0/0) | 0(0/0) | 0(0/0) | 0(0/0) | 0(0/0) | 12(5/7) |
| WIY31 | 8(6/2) | 0(0/0) | 12(3/9) | 6(0/6) | 0(0/0) | 0(0/0) | 0(0/0) | 0(0/0) | 6(6/0) | 0(0/0) | 0(0/0) | 0(0/0) | 0(0/0) | 0(0/0) | 2(0/2) | 34(15/19) |
| WEY7 | 2(2/0) | 4(4/0) | 0(0/0) | 2(0/2) | 0(0/0) | 0(0/0) | 0(0/0) | 0(0/0) | 0(0/0) | 0(0/0) | 0(0/0) | 0(0/0) | 0(0/0) | 0(0/0) | 0(0/0) | 8(6/2) |
| WDY23 | 0(0/0) | 0(0/0) | 0(0/0) | 0(0/0) | 13(0/13) | 0(0/0) | 0(0/0) | 0(0/0) | 6(2/4) | 6(6/0) | 0(0/0) | 0(0/0) | 0(0/0) | 0(0/0) | 0(0/0) | 25(8/17) |
| WEB25 | 0(0/0) | 0(0/0) | 3(3/0) | 0(0/0) | 0(0/0) | 0(0/0) | 0(0/0) | 0(0/0) | 0(0/0) | 0(0/0) | 0(0/0) | 0(0/0) | 0(0/0) | 0(0/0) | 0(0/0) | 3(3/0) |
| WEB26 | 0(0/0) | 0(0/0) | 0(0/0) | 0(0/0) | 13(0/13) | 0(0/0) | 0(0/0) | 0(0/0) | 6(2/4) | 6(6/0) | 0(0/0) | 0(0/0) | 0(0/0) | 0(0/0) | 0(0/0) | 25(8/17) |
| Subtotal | 29(18/11) | 14(8/6) | 24(15/9) | 37(10/27) | 27(7/20) | 12(7/5) | 8(7/1) | 5(3/2) | 36(20/16) | 53(30/23) | 0(0/0) | 26(5/21) | 18(15/3) | 3(3/0) | 30(16/14) | 322(164/158) |
| *Rhizobium* *genosp.* Ⅰ (Clade2,C2) (0.98%, 6 isolates, 1 IGS types, 1 sites) | | | | | | | | | | | | | | | | |
| WMY6 | 0(0/0) | 0(0/0) | 0(0/0) | 0(0/0) | 0(0/0) | 6(2/4) | 0(0/0) | 0(0/0) | 0(0/0) | 0(0/0) | 0(0/0) | 0(0/0) | 0(0/0) | 0(0/0) | 0(0/0) | 6(2/4) |
| Subtotal | 0(0/0) | 0(0/0) | 0(0/0) | 0(0/0) | 0(0/0) | 6(2/4) | 0(0/0) | 0(0/0) | 0(0/0) | 0(0/0) | 0(0/0) | 0(0/0) | 0(0/0) | 0(0/0) | 0(0/0) | 6(2/4) |
| *Rhizobium genosp.* Ⅱ (Clade3,C3) (1.62%, 10 isolates,1 IGS types, 3 sites) | | | | | | | | | | | | | | | | |
| WFY14 | 0(0/0) | 0(0/0) | 0(0/0) | 0(0/0) | 0(0/0) | 0(0/0) | 4(2/2) | 3(3/0) | 3(3/0) | 0(0/0) | 0(0/0) | 0(0/0) | 0(0/0) | 0(0/0) | 0(0/0) | 10(8/2) |
| Subtotal | 0(0/0) | 0(0/0) | 0(0/0) | 0(0/0) | 0(0/0) | 0(0/0) | 4(2/2) | 3(3/0) | 3(3/0) | 0(0/0) | 0(0/0) | 0(0/0) | 0(0/0) | 0(0/0) | 0(0/0) | 10(8/2) |
| *Rhizobium hidalgonense* (Clade4,C4) (2.8%, 17 isolates, 2 IGS types, 2 sites) | | | | | | | | | | | | | | | | |
| WFB6 | 0(0/0) | 0(0/0) | 0(0/0) | 0(0/0) | 0(0/0) | 0(0/0) | 0(0/0) | 0(0/0) | 0(0/0) | 0(0/0) | 0(0/0) | 11(6/5) | 0(0/0) | 0(0/0) | 0(0/0) | 11(6/5) |
| WFY21 | 0(0/0) | 0(0/0) | 0(0/0) | 0(0/0) | 0(0/0) | 0(0/0) | 0(0/0) | 0(0/0) | 0(0/0) | 0(0/0) | 0(0/0) | 0(0/0) | 0(0/0) | 6(5/1) | 0(0/0) | 6(5/1) |
| Subtotal | 0(0/0) | 0(0/0) | 0(0/0) | 0(0/0) | 0(0/0) | 0(0/0) | 0(0/0) | 0(0/0) | 0(0/0) | 0(0/0) | 0(0/0) | 11(6/5) | 0(0/0) | 6(5/1) | 0(0/0) | 17(11/6) |
| *Rhizobium binae* (Clade5,C5) (6.3%, 39 isolates, 1 IGS type, 4 sites) | | | | | | | | | | | | | | | | |
| WMB11 | 10(0/10) | 13(5/8) | 0(0/0) | 8(0/8) | 8(8/0) | 0(0/0) | 0(0/0) | 0(0/0) | 0(0/0) | 0(0/0) | 0(0/0) | 0(0/0) | 0(0/0) | 0(0/0) | 0(0/0) | 39(13/26) |
| Subtotal | 10(0/10) | 13(5/8) | 0(0/0) | 8(0/8) | 8(8/0) | 0(0/0) | 0(0/0) | 0(0/0) | 0(0/0) | 0(0/0) | 0(0/0) | 0(0/0) | 0(0/0) | 0(0/0) | 0(0/0) | 39(13/26) |
| *Rhizobium bangladeshense* (Clade6,C6) (3.7%, 23 isolates, 2 IGS types, 5 site) | | | | | | | | | | | | | | | | |
| WNB8 | 0(0/0) | 0(0/0) | 0(0/0) | 0(0/0) | 0(0/0) | 9(0/9) | 0(0/0) | 0(0/0) | 0(0/0) | 0(0/0) | 0(0/0) | 0(0/0) | 0(0/0) | 0(0/0) | 0(0/0) | 9(0/9) |
| WNY29 | 0(0/0) | 0(0/0) | 0(0/0) | 0(0/0) | 0(0/0) | 0(0/0) | 3(2/1) | 3(3/0) | 5(0/5) | 3(1/2) | 0(0/0) | 0(0/0) | 0(0/0) | 0(0/0) | 0(0/0) | 14(6/8) |
| Subtotal | 0(0/0) | 0(0/0) | 0(0/0) | 0(0/0) | 0(0/0) | 9(0/9) | 3(2/1) | 3(3/0) | 5(0/5) | 3(1/2) | 0(0/0) | 0(0/0) | 0(0/0) | 0(0/0) | 0(0/0) | 23(6/17) |
| *Rhizobium genosp.* Ⅲ (Clade7,C7) (1.1%, 7 isolates, 1 IGS type, 1 site) | | | | | | | | | | | | | | | | |
| WKY7 | 0(0/0) | 7(0/7) | 0(0/0) | 0(0/0) | 0(0/0) | 0(0/0) | 0(0/0) | 0(0/0) | 0(0/0) | 0(0/0) | 0(0/0) | 0(0/0) | 0(0/0) | 0(0/0) | 0(0/0) | 7(0/7) |
| Subtotal | 0(0/0) | 7(0/7) | 0(0/0) | 0(0/0) | 0(0/0) | 0(0/0) | 0(0/0) | 0(0/0) | 0(0/0) | 0(0/0) | 0(0/0) | 0(0/0) | 0(0/0) | 0(0/0) | 0(0/0) | 7(0/7) |
| *Rhizobium anhuiense* (Clade8,C8) (31%, 191 isolates, 14 IGS types, 14 sites) | | | | | | | | | | | | | | | | |
| WGB14 | 5(0/5) | 0(0/0) | 0(0/0) | 0(0/0) | 0(0/0) | 0(0/0) | 0(0/0) | 0(0/0) | 0(0/0) | 0(0/0) | 6(0/6) | 0(0/0) | 0(0/0) | 0(0/0) | 0(0/0) | 11(0/11) |
| WGY25 | 5(3/2) | 0(0/0) | 0(0/0) | 2(1/1) | 0(0/0) | 0(0/0) | 0(0/0) | 0(0/0) | 0(0/0) | 0(0/0) | 0(0/0) | 0(0/0) | 0(0/0) | 0(0/0) | 0(0/0) | 7(4/3) |
| WHB5 | 0(0/0) | 0(0/0) | 0(0/0) | 0(0/0) | 0(0/0) | 0(0/0) | 0(0/0) | 0(0/0) | 0(0/0) | 0(0/0) | 0(0/0) | 0(0/0) | 0(0/0) | 4(3/1) | 0(0/0) | 4(3/1) |
| WHY31 | 0(0/0) | 0(0/0) | 0(0/0) | 0(0/0) | 0(0/0) | 0(0/0) | 0(0/0) | 0(0/0) | 0(0/0) | 1(1/0) | 0(0/0) | 0(0/0) | 0(0/0) | 0(0/0) | 0(0/0) | 1(1/0) |
| WKY15 | 0(0/0) | 2(0/2) | 3(0/3) | 0(0/0) | 0(0/0) | 0(0/0) | 0(0/0) | 0(0/0) | 0(0/0) | 0(0/0) | 0(0/0) | 0(0/0) | 0(0/0) | 0(0/0) | 0(0/0) | 5(0/5) |
| WOY25 | 0(0/0) | 0(0/0) | 0(0/0) | 0(0/0) | 0(0/0) | 0(0/0) | 0(0/0) | 0(0/0) | 0(0/0) | 0(0/0) | 0(0/0) | 0(0/0) | 0(0/0) | 5(0/5) | 0(0/0) | 5(0/5) |
| WNY22 | 0(0/0) | 0(0/0) | 0(0/0) | 0(0/0) | 0(0/0) | 0(0/0) | 0(0/0) | 0(0/0) | 0(0/0) | 0(0/0) | 0(0/0) | 0(0/0) | 7(4/3) | 0(0/0) | 0(0/0) | 7(4/3) |
| WGY20 | 0(0/0) | 0(0/0) | 0(0/0) | 0(0/0) | 0(0/0) | 0(0/0) | 0(0/0) | 0(0/0) | 0(0/0) | 0(0/0) | 0(0/0) | 0(0/0) | 10(5/5) | 0(0/0) | 0(0/0) | 10(5/5) |
| WKB22 | 0(0/0) | 0(0/0) | 0(0/0) | 0(0/0) | 0(0/0) | 0(0/0) | 0(0/0) | 0(0/0) | 0(0/0) | 0(0/0) | 0(0/0) | 17(14/3) | 0(0/0) | 0(0/0) | 0(0/0) | 17(14/3) |
| WFB12 | 0(0/0) | 0(0/0) | 0(0/0) | 0(0/0) | 0(0/0) | 0(0/0) | 0(0/0) | 3(2/1) | 4(2/2) | 0(0/0) | 0(0/0) | 0(0/0) | 0(0/0) | 6(0/6) | 0(0/0) | 13(4/9) |
| WAY18 | 0(0/0) | 0(0/0) | 0(0/0) | 0(0/0) | 0(0/0) | 0(0/0) | 12(7/5) | 9(0/9) | 3(0/3) | 0(0/0) | 0(0/0) | 0(0/0) | 0(0/0) | 3(0/3) | 0(0/0) | 27(7/20) |
| WAY13 | 24(21/3) | 0(0/0) | 0(0/0) | 5(0/5) | 6(0/6) | 0(0/0) | 0(0/0) | 12(12/0) | 0(0/0) | 0(0/0) | 12(12/0) | 0(0/0) | 0(0/0) | 0(0/0) | 0(0/0) | 59(45/14) |
| WAB15 | 0(0/0) | 0(0/0) | 0(0/0) | 0(0/0) | 0(0/0) | 0(0/0) | 12(7/5) | 9(0/9) | 3(0/3) | 0(0/0) | 0(0/0) | 0(0/0) | 0(0/0) | 3(0/3) | 0(0/0) | 27(7/20) |
| WAB17 | 24(21/3) | 0(0/0) | 0(0/0) | 5(0/5) | 6(0/6) | 0(0/0) | 0(0/0) | 12(12/0) | 0(0/0) | 0(0/0) | 12(12/0) | 0(0/0) | 0(0/0) | 0(0/0) | 0(0/0) | 59(45/14) |
| WGB17 | 0(0/0) | 0(0/0) | 0(0/0) | 0(0/0) | 0(0/0) | 6(2/4) | 10(10/0) | 0(0/0) | 0(0/0) | 0(0/0) | 0(0/0) | 0(0/0) | 0(0/0) | 0(0/0) | 0(0/0) | 16(12/4) |
| WHY29 | 0(0/0) | 0(0/0) | 0(0/0) | 0(0/0) | 0(0/0) | 3(3/0) | 0(0/0) | 0(0/0) | 6(0/6) | 0(0/0) | 0(0/0) | 0(0/0) | 0(0/0) | 0(0/0) | 0(0/0) | 9(3/6) |
| Subtotal | 34(24/10) | 2(0/2) | 3(0/3) | 7(1/6) | 6(0/6) | 9(5/4) | 22(17/5) | 24(14/10) | 13(2/11) | 1(1/0) | 18(12/6) | 17(14/3) | 17(9/8) | 18(3/15) | 0(0/0) | 191(102/89) |
| Total number of isolates and the number of peaB/peaY | 73(42/31) | 36(13/23) | 27(15/12) | 52(11/41) | 41(15/26) | 36(14/22) | 37(28/19) | 35(23/12) | 57(25/32) | 57(32/25) | 18(12/6) | 54(25/29) | 35(24/11) | 27(11/16) | 30(16/14) | 615(306/309) |

^a^ YC-MD, Mouding County, Chuxiong city; YC-YA, Yaoan county, Chuxiong city; YC-DY, Dayao county, Chuxiong city; YC-LF, Lufeng city, Chuxiong city; YC-DH, Donghua town, Chuxiong city; YC-WD, Wuding County, Chuxiong city; YC-SP, Shuangpai county, Chuxiong city; YC-NH, Nanhua county, Chuxiong city; YD-XY, Xiangyun county, Dali city; YD-WS, Weishan county, Dali city; YD-MD, Midu county, Dali city; YD-EY, Eryuan county, Dali city; YB-LY, Longyang district, Baoshan city; YB-CN, Changning county, Baoshan city; YB-SD, Shidian county, Baoshan city;

**TABLE S5** Detection of IAA producing ability of rhizobia

| Representative strain (no.) | IAA production (mg/L) | Representative strain (no.) | IAA production (mg/L) | Representative strain (no.) | IAA production (mg/L) |
| --- | --- | --- | --- | --- | --- |
| WAB15 | 4.812±0.263 jkl | WFB6 | 2.026±0.387 uvw | WJY1 | 4.159±0.346 lmno |
| WAB17 | 7.188±0.360 f | WFY14 | 3.154±0.336 pqr | WJY16 | 6.094±0.292 gh |
| WAY13 | 5.171±0.411 ijk | WFY21 | 2.812±0.282 rst | WJY2 | 6.009±0.442 gh |
| WAY18 | 3.598±0.385 opq | WGB14 | 8.761±0.360 de | WKB22 | 3.991±0.231 mno |
| WBY2 | 3.137±0.341 pqr | WGB17 | 3.581±0.313 opq | WKY15 | 4.829±0.194 jkl |
| WBY27 | 0.453±0.030 y | WGY20 | 5.068±0.436 ijk | WKY7 | 9.462±0.286 c |
| WCB13 | 11.462±0.769 b | WGY25 | 5.650±0.392 hi | WLB11 | 4.231±0.438 lmno |
| WCB18 | 64.556±0.465 a | WHB5 | 8.214±0.282 e | WLB27 | 2.726±0.341 rstu |
| WCY11 | 9.137±0.341 cd | WHY29 | 4.214±0.309 lmno | WLB6 | 4.282±0.256 lmno |
| WDB13 | 5.256±0.308 ijk | WHY31 | 2.248±0.334 tuv | WMB11 | 2.675±0.442 rstu |
| WDY12 | 2.094±0.349 uvw | WIB14 | 2.162±0.309 tuvw | WMY6 | 2.214±0.385 tuv |
| WDY2 | 2.304±0.275 stu | WIB31 | 2.949±0.470 qrs | WNB8 | 0.453±0.030 y |
| WDY23 | 3.655±0.385 nop | WIY23 | 5.444±0.805 hij | WNY22 | 5.085±0.462 ijk |
| WEB25 | 6.316±0.258 g | WIY31 | 4.829±0.258 jkl | WNY29 | 1.598±0.360 vwx |
| WEB26 | 1.496±0.078 wx | WJB19 | 4.641±0.470 klm | WOY25 | 2.299±0.334 stu |
| WFB12 | 0.983±0.207 xy | WJB4 | 4.350±0.385 lmn |  |  |

The results of three repeated experiments for each treatment were statistically analyzed. Data are represented as mean ± standard deviation, The difference was significant by 0.05.

**TABLE S6 Representative strains with the highest abiotic stress tolerances**

| Strains | Tolerance to | | | | |
| --- | --- | --- | --- | --- | --- |
|  | acidity | alkalinity | NaCl | PEG | glyphosate |
| WCB18 | 6 | 11 | 4% | 3% | 1.8% |
| WBY27 | 6 | 10 | 4% | 5% | 1.8% |
| WBY2 | 6 | 11 | 4% | 0% | 1.8% |
